# Rationally Designed Novel Phenyloxazoline Synthase Inhibitors: Chemical Synthesis and Biological Evaluation to Accelerate the Discovery of New Antimycobacterial Antibiotics

**DOI:** 10.3390/molecules28248115

**Published:** 2023-12-15

**Authors:** Mousumi Shyam, Gourab Bhattacharje, Chris Daniel, Amrendra Kumar, Pragya Yadav, Piyali Mukherjee, Samsher Singh, Amit Kumar Das, Tadigoppula Narender, Amit Singh, Venkatesan Jayaprakash, Sanjib Bhakta

**Affiliations:** 1 Department of Pharmaceutical Sciences & Technology, Birla Institute of Technology, Mesra, Ranchi 835215, India; mousumishyam48@gmail.com; 2 Mycobacteria Research Laboratory, School of Natural Sciences, Institute of Structural and Molecular Biology, Birkbeck, University of London, Malet Street, London WC1E 7HX, UK; cdanie10@student.bbk.ac.uk; 3 Department of Biotechnology, Indian Institute of Technology Kharagpur, Kharagpur 721302, India; gourab.bhattacharje@gmail.com (G.B.); amitk@bt.iitkgp.ac.in (A.K.D.); 4 Division of Medicinal & Process Chemistry, CSIR-Central Drug Research Institute, Sector 10 Janakipuram Extension, Sitapur Road, Lucknow 226031, India; amrendra086@gmail.com (A.K.); pragyayadav8918@gmail.com (P.Y.); t_narendra@cdri.res.in (T.N.); 5 Department of Microbiology and Cell Biology, Centre for Infectious Disease Research, Indian Institute of Science, CV Raman Avenue, Bengaluru 560012, India; piyalibioc@gmail.com (P.M.); sambio.singh@gmail.com (S.S.); asingh@iisc.ac.in (A.S.)

**Keywords:** mycobactin metabolism, phenyloxazoline synthase, cyclization domain, transition-state analogues, MD simulations, MMPBSA studies, intracellular killing, efflux pump

## Abstract

The uncontrolled spread of drug-resistant tuberculosis (DR-TB) clinical cases necessitates the urgent discovery of newer chemotypes with novel mechanisms of action. Here, we report the chemical synthesis of rationally designed novel transition-state analogues (TSAs) by targeting the cyclization (Cy) domain of phenyloxazoline synthase (MbtB), a key enzyme of the conditionally essential siderophore biosynthesis pathway. Following bio-assay-guided evaluation of TSA analogues preferentially in iron-deprived and iron-rich media to understand target preferentiality against a panel of pathogenic and non-pathogenic mycobacteria strains, we identified a hit, i.e., **TSA-5**. Molecular docking, dynamics, and MMPBSA calculations enabled us to comprehend **TSA-5**’s stable binding at the active site pocket of **MbtB_Cy** and the results imply that the **MbtB_Cy** binding pocket has a strong affinity for electron-withdrawing functional groups and contributes to stable polar interactions between enzyme and ligand. Furthermore, enhanced intracellular killing efficacy (8 μg/mL) of **TSA-5** against *Mycobacterium aurum* in infected macrophages is noted in comparison to moderate in vitro antimycobacterial efficacy (64 μg/mL) against *M. aurum*. **TSA-5** also demonstrates whole-cell efflux pump inhibitory activity against *Mycobacterium smegmatis*. Identification of **TSA-5** by focusing on the modular **MbtB_Cy** domain paves the way for accelerating novel anti-TB antibiotic discoveries.

## 1. Introduction

Alarmingly, emerging drug-resistant strains of *Mycobacterium tuberculosis* (Mtb) (multidrug-resistant (MDR) Mtb, extensively drug-resistant (XDR) Mtb) are resulting in uncontrolled drug resistance in tuberculosis (TB) treatment [1,2]. In 2021, Mtb infected approximately 10 million people and led to more than 1.6 million deaths due to TB [1]. Unfortunately, MDR Mtb strains are resistant to the most effective first-line anti-TB therapeutics, i.e., rifampicin (RIF) and isoniazid (INH), whereas XDR Mtb strains are resistant to all first- and second-line anti-TB drugs, as well as fluoroquinolones (FQs) [1]. The recent World Health Organization (WHO) TB report highlighted that nearly one in every four people worldwide has a debilitating chronic TB infection, with 202,000 showing RIF-resistant TB (RR TB) [1,3]. To overcome this dismal drug resistance scenario, we are in urgent need of next-generation therapeutics, which may be developed by identifying novel chemotypes with alternative mechanisms of action to accelerate the anti-TB drug discovery and replenish the antibiotic regimen against TB.

In this context, the conditionally essential siderophore-mediated iron transportation machinery uniquely conserved in mycobacteria provides an encouraging and yet underexplored avenue for accelerating TB chemotherapeutic discoveries [4,5,6,7]. Mycobacteria encounter iron scarcity in the host macrophages and that iron-stress environment instigates mycobacteria to produce salicyl-capped siderophores, mycobactin/carboxymycobactin (MB/CMB). MB/CMB coordinate chelation and internalization of essential micronutrient iron from host iron-binding proteins via a complex machinery to support mycobacteria’s smooth survival in the host immune system [6,7]. The siderophore biosynthesis pathway is commonly shared by pathogenic, nontuberculous, and non-pathogenic mycobacteria, i.e., Mtb, *M. abscessus*, *M. neoaurum*, and *M. smegmatis* [6,7]. Siderophore biosynthesis is tightly regulated by fourteen conditionally essential genes (*mbtA*-*mbtN*) [6,7]. Amongst these fourteen essential enzymes, two key enzymes, i.e., MbtA and MbtI, have received major attention from the scientific community in the past decades for siderophore biosynthesis inhibitor discovery, and lackluster effort has been given to the exploration of other enzymes as prospective drug targets for novel antibiotic discoveries. 

**Figure 1:**
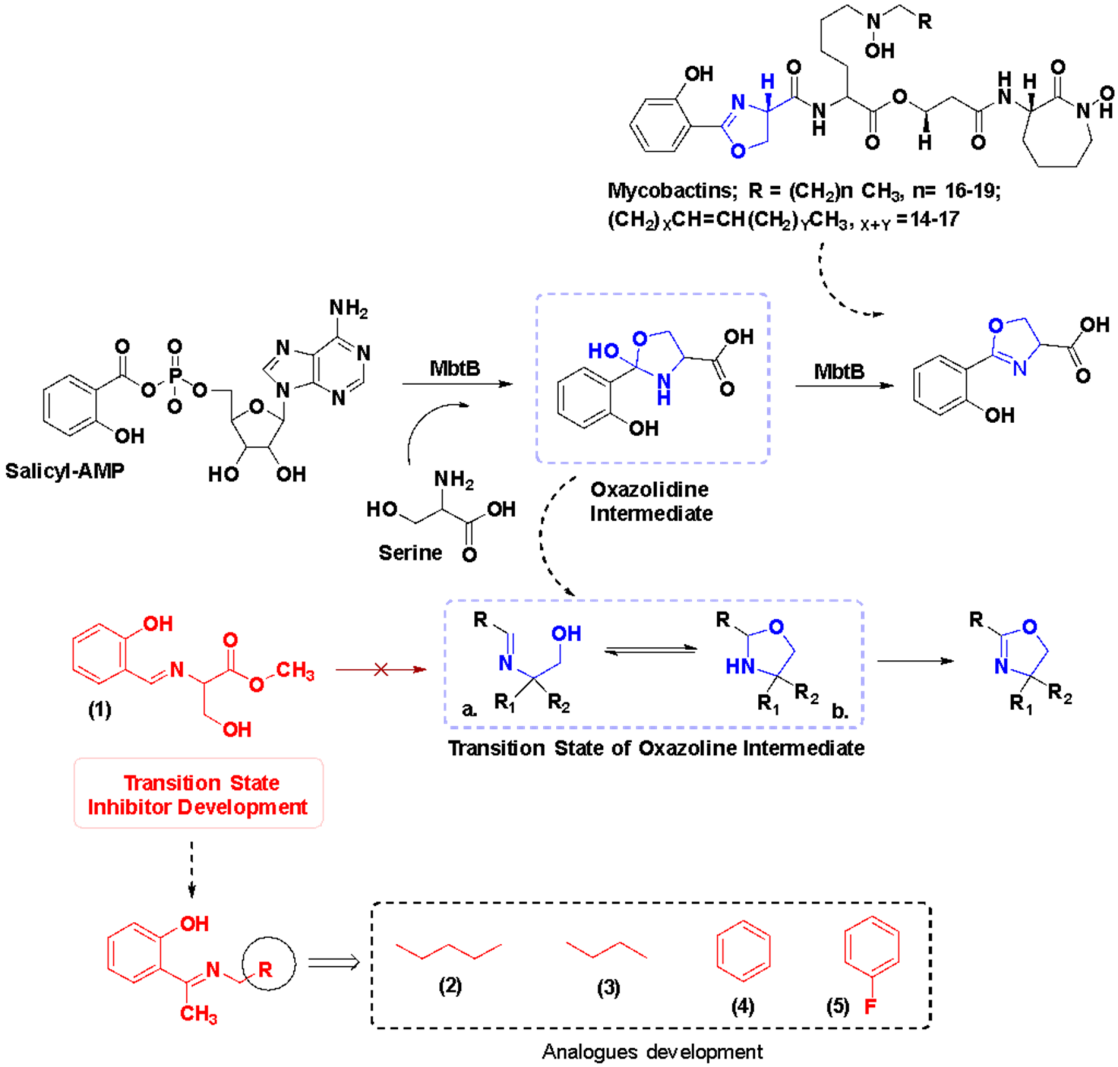
MbtB domain oversees the heterocyclic oxazolidine ring formation. **MbtB_Cy** domain catalyzes a condensation reaction between a peptidyl thioester intermediate and a more recent threonine extender and results in a new amide bond formation. In the next step, a nucleophilic attack on the carbonyl carbon of the amide bond is executed by the hydroxyl or thiol group found in the side chain. Finally, the newly formed hemiaminal undergoes a dehydration reaction and results in the heterocyclic oxazoline ring formation. By mimicking the oxazoline intermediate, transition-state (a. and b.) analogues (TSAs) are designed. TSAs’ or intermediate mimics’ development follows conventional drug discovery approaches that were used previously for the siderophore biosynthesis inhibitors’ development, i.e., Salicyl-AMP and Salicyl-AMP analogues (bonafide MbtA inhibitors) [6,7].

In the siderophore biosynthesis pathway, phenyloxazoline synthase (MbtB) oversees heterocyclic oxazoline nucleus incorporation in the mycobactin mega-architecture [4,8]. MbtB itself is a multi-modular enzyme comprising acyl carrier protein (ACP), adenylation (A), cyclization (Cy), peptidyl carrier protein (PCP), and thioesterase (TE) subdomains with distinct catalytic functionality, and particularly, the Cy domain overseas a distinct Pictet–Spengler heterocyclization reaction to form a five-membered 5-methyloxazoline ring [9], which provides structural rigidity to the peptide. The study reported by De Voss et al. [10] highlighted that the *mbtB* gene replacement with a hygromycin-resistance cassette resulted in restricted Mtb growth in the iron-deficient condition. This study highlighted the conditional essentiality of the *mbtB* gene for the survival of Mtb in an iron-deprived condition for the very first time [10]. Considering significant functional versatility and a central catalytic role, we hypothesized that the **MbtB_Cy** domain represents an unexplored novel drug target for the discovery of next-generation antibiotics against TB. To the best of our knowledge, there are no reports on any inhibitors or structural insights into **MbtB_Cy**. In continuation with our previous efforts and expert opinions on siderophore biosynthesis inhibitor discoveries as the next-generation TB therapeutic [7,11,12,13], we thought to shed light on the rationale for designing (Figure 1) novel transition-state analogues (TSAs) as a possible new HIT by targeting the MbtB enzyme.

**Scheme 1:**
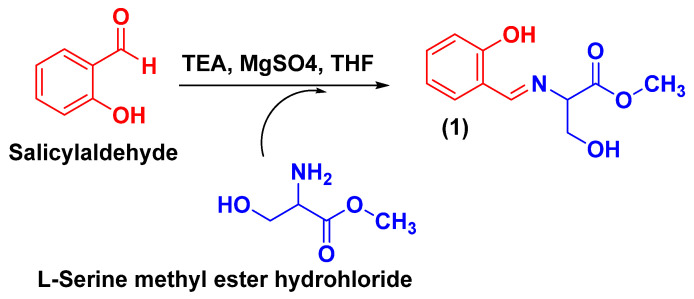
Chemical synthesis of **TSA-1**.

**Scheme 2:**
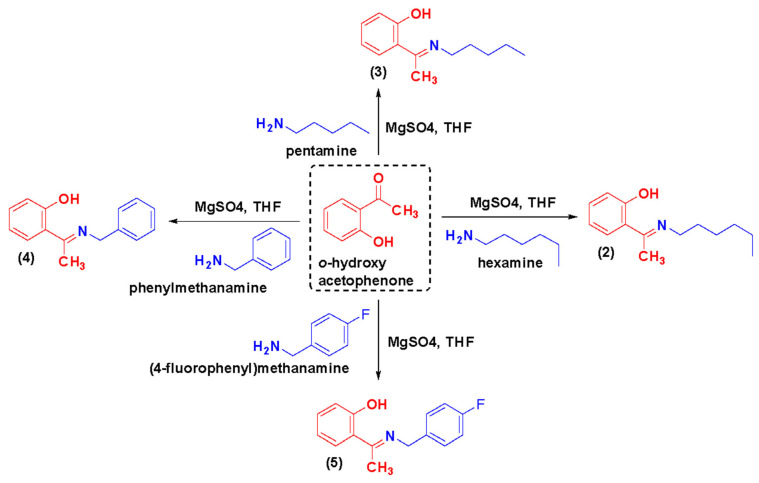
Chemical synthesis of **TSA-(2–5)**.

In this study, we integrated a whole-cell phenotypic screening against different strains of mycobacteria to investigate the antimycobacterial activity of rationally designed, chemically synthesized (Scheme 1 and Scheme 2) TSA analogues and an identified HIT. Additionally, SPOTi-based screening against intracellular surviving mycobacteria was performed because Mtb’s ability to survive and proliferate inside macrophages and its waxy mycobacterial envelope permeability are two key features that contribute significantly to the pathogenesis as well as treatment difficulties [14]. It is necessary that inhibitors must penetrate and access the intracellular compartment niche of macrophages to exert a lethal impact on the intracellularly surviving bacilli to mitigate the unprecedented failure of drug candidates at the later stage of clinical trials [14]. Next, the cytotoxicity profile of the HIT candidate was investigated against eucaryotic cell lines. Subsequently, construction of the **MbtB_Cy** homology model and in silico enzyme–ligand interaction investigation through molecular docking, molecular dynamics simulation, and MMPBSA calculations were taken into consideration to identify the stable binding orientations and significant interactions between the HIT candidate and **MbtB_Cy**. Additionally, a whole-cell efflux pump assay was also conducted to investigate the efflux pump inhibition profile of the HIT candidate, to remove the burden of drug efflux-pump-mediated resistance.

## 2. Results and Discussion

### 2.1. Chemical Synthesis and Characterization of TSA

TSAs are chemically azomethine compounds consisting of an *o*-hydroxyphenyl unit with a bidentate ligand feature. Scheme 1 and Scheme 2 illustrate the synthetic strategy for the preparation of the designed azomethine analogues. **TSA-1** was synthesized from the condensation of salicylaldehyde and L-serine methyl ester hydrochloride in the nitrogen environment. The derivatives **TSA-(2–5)** were synthesized by condensing *o*-hydroxy acetophenone with different amines in the nitrogen environment. The characterization of newly synthesized analogues was carried out using ^1^H-NMR, ^13^C-NMR, HRMS, DEPT-135, and DEPT-90 (Supporting Information: ^1^H-NMR, ^13^C-NMR, HRMS, DEPT-135, and DEPT-90 spectra).

*Methyl (E)-3-hydroxy-2-(((2-hydroxyphenyl)imino)methyl) Propanoate* (**TSA-1**). Dark yellow, semi-solid, and with a yield of 89%. Mol. formula: C_11_H_13_NO_4_; Mol. wt.: 223; M.P: 171.1 °C; ^1^H NMR (400 MHz, CDCl_3_) δ (ppm) 3.64 (s, -CH_3_, 3H), 3.84 (s, CH_2_, 1H), 4.021 (d, -CH_2_, 2H), 6.79–7.26 (m, Ar-H, 4H), 8.36 (s, CH, 1H), 9.79 (s, OH, 1H), 12.68 (s, OH, 1H); ^13^C NMR (100 MHz, CDCl_3_) δ (ppm) 170.30, 169.59, 160.89, 137.09, 133.77, 132.96, 131.58, 120.68, 118.35, 117.53, 152.10, 77.15, 72.67, 66.51; ESI-MS (*m*/*z*): 224 (M+1)^+^; DEPT-135 (100 MHz, CDCl_3_) 67.5 -CH_2_; DEPT-90 (100 MHz, CDCl_3_) 168.65, 133.76, 132.04, 119.87, 117.60, 72.64 (-CH); HRMS (ESI) calcd. for C_11_H_13_NO_4_ (M+H^+^) 224.23, found 224.09. 

*(E)-2-(1-(pentylimino)ethyl)phenol* (**TSA-2**). Brown, semi-solid, and with a yield of 87%. Mol. formula: C_13_H_19_NO; Mol. wt.: 205.15; M.P: 141–143 °C; ^1^H NMR (400 MHz, CDCl_3_) δ (ppm) 1.34 (s, -CH_3_, 3H), 1.4016 and 1.4471 (d, -2CH_2_, 4H), 1.77 (d, -CH_2_, 2H), 2.29 (s, CH_3_, 3H), 3.50 (s, CH_2_, 1H), 6.68–7.47 (m, Ar-H, 4H), 16.89 (s, OH, 1H); ^13^C NMR (100 MHz, CDCl_3_) δ (ppm) 171.46, 165.62, 132.63, 127.95, 119.03, 116.32, 77.12, 48.66, 29.73, 22.45, 14.00; HRMS (ESI) calcd. for C_13_H_19_NO (M+H^+^) 206.15, found 206.15.

*(E)-2-(1-(hexylimino)ethyl)phenol* (**TSA-3**). Yellow, semi-solid, and with a yield of 85%. Mol. formula: C_14_H_21_NO; Mol. wt.: 219.33; M.P: 165–167 °C; ^1^H NMR (400 MHz, CDCl_3_) δ (ppm) 0.9166 (s, -CH_3_, 3H), 1.286 and 1.311 (d, 2CH_2_, 4H), 1.339 (d, -CH_2_, 2H), 1.7 (d, -CH_2_, 2H), 1.963 (s, CH_3_, 3H), 3.56 (s, CH_2_, 1H), 6.70–7.50 (m, Ar-H, 4H); ^13^C NMR (100 MHz, CDCl_3_) δ (ppm) 171.41, 165.59, 132.66, 127.91, 119.06, 116.35, 77.03, 48.72, 31.57, 30.24, 27.06, 22.56, 14.05; HRMS (ESI) calcd. for C_14_H_21_NO (M+H^+^) 220.16, found 220.1693. 

*(E)-2-(1-(benzyl)imino)ethyl) Phenol* (**TSA-4**). Yellow mass with a yield of 89%. Mol. formula: C_15_H_15_NO; Mol. wt.: 225.12; M.P: 119–120 °C; ^1^H NMR (400 MHz, CDCl_3_) δ (ppm) 2.4191 (s, -CH_3_, 3H), 4.8067 (s, CH_2_, 2H), 6.772–7.565 (m, Ar-H, 4H), 16.326 (s, OH, 1H); ^13^C NMR (100 MHz, CDCl_3_) δ (ppm) 172.27, 163.88, 138.42, 132.62, 128.77, 128.09, 127.45, 127.23, 119.41, 118.77, 117.15, 77.03, 53.35, 14.70; HRMS (ESI) calcd. for C_15_H_15_NO (M+H^+^) 220.12, found 220.1220.

(*E)-2-(1-((4-fluorobenzyl)imino)ethyl) Phenol* (**TSA-5**). Yellow mass with a yield of 91%. Mol. formula: C_15_H_14_FNO; Mol. wt.: 243; M.P: 58–60 °C; ^1^H NMR (400 MHz, CDCl_3_) δ (ppm) 2.4147 (s, -CH_3_, 3H), 4.7575 (s, CH_2_, 2H), 6.7840–7.5667 (m, Ar-H, 4H), 16.1403 (s, OH, 1H); ^13^C NMR (100 MHz, CDCl_3_) δ (ppm) 172.28, 163.38, 134.31, 132.65, 129.11, 129.03, 128.11, 119.44, 118.65, 117.35, 115.70, 115.49, 77.04, 52.75, 14.75; HRMS (ESI) calcd. for C_15_H_15_NO (M+H^+^) 244.28, found 244.1127.

### 2.2. Antimycobacterial Activity Investigations and HIT Identification from the Structure–Activity Relationship (SAR)

Novel TSA analogues were evaluated for their antimycobacterial activity against a panel of pathogenic and non-pathogenic mycobacterial species, such as pathogenic Mtb H37Rv, non-pathogenic intracellularly surviving *M. aurum*, non-pathogenic fast-growing *M. smegmatis* mc^2^ 155, and nontuberculous *M. abscessus* NCTC 13031 (Table 1). The determination of minimum inhibitory concentrations (MIC_90_) in both iron-rich (GAS-Fe) and iron-deprived (GAS) media was performed, following an earlier reported protocol [11,12]. The target preferentiality of each compound was determined by considering two different growth environments, iron-rich (GAS-Fe) and iron-deprived (GAS) media, and the result was denoted as the target selectivity index (TSI; ratio of MIC_90_ in iron-rich medium to MIC_90_ in iron-deprived medium), which is distinctly different from the selectivity index (SI, where the SI value represents the ratio of growth inhibitory concentration (GIC) to the pathogen’s MIC). The only active candidate in this pilot library was discovered to be **TSA-5**, which had a *para*-fluoro substituted phenyl ring on the amino terminus. **TSA-5** demonstrated MIC_90_ values in GAS and GAS-Fe medium of 32 and >256 µg/mL, respectively, indicating a TSI value of >8, which gives an insight into the antimycobacterial efficacy resulting from mycobactin synthesis suppression. This investigation uncovered an intriguing finding that if we closely examined the chemical structures of compounds **TSA-4** and **TSA-5**, the only variation in the substitutions was a *para*-F atom embellished on phenyl ring B of **TSA-5**, which affected the antitubercular activity. The F-atom has been shown to be the most favorable halogenic substituent for antitubercular activity in earlier research [15,16], and this study also found a similar phenomenon, demonstrating the relationship between pleiotropic chemotype influences and the complimentary activity of **TSA-5**. 

### 2.3. Molecular Docking, Molecular Dynamics (MD) Simulation, and MMPBSA Analysis to Validate the Putative Drug Target of **TSA-5**

**Figure 2:**
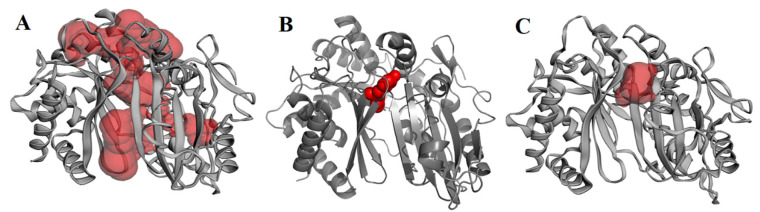
Prediction of active site pocket of **MbtB_Cy** and comparison with the docked complex of **TSA-5**. (**A**) Surface area of the largest pocket precited was 1839.06 Å^2^. (**B**) Docked structure obtained from AutoDock4.0 shows that **TSA-5** fits into the predicted pocket of **MbtB_Cy**. (**C**) Further analysis of all the predicted pockets showed that **TSA-5** was docked close to pocket 4 (surface area 113.47 Å^2^).

**Figure 3:**
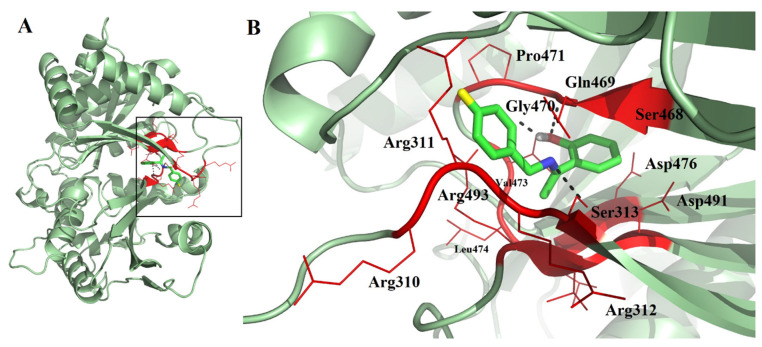
(**A**) The homology model of the **MbtB_Cy** domain was docked with **TSA-5**, which indicated a stable binding. (**B**) Interactions of **TSA-5** in the docked complex suggested that Arg310, Arg311, Arg312, Ser313, Gln469, Gly470, Pro471, Val473, Leu474, Asp476, and Arg493 of **MbtB_Cy** play critical roles.

To understand the possible interactions of **TSA-5** with the cyclization domain of MbtB (**MbtB_Cy**), and validate the drug-design hypothesis, detailed in silico investigations were performed. The lack of an X-ray crystallography structure prompted us to create a homology model of our protein of interest, **MbtB_Cy**, using SWISS-MODEL [17]. The homology model was further validated by the Ramachandran plot [18] (Supplementary Figure S1). To predict the binding pocket for further molecular docking analyses, we attempted to search for the possible binding pockets of **MbtB_Cy**. We analyzed the predicted binding pockets of **MbtB_Cy** by using the Computed Atlas of Surface Topography of protein CASTp 3.0 [19] (Figure 2), and the tunnel-shaped multicomponent active pocket was observed to be 1839.06 Å, encompassing a large part of the protein. Molecular docking analysis of **TSA-5** was performed using Autodock 4.2 [20], where the grid covered the whole tunnel-shaped pocket of **MbtB_Cy**. The lowest free energy of binding between **MbtB_Cy** and **TSA-5** was observed to be −8.50 kcal/mol. Analyzing the docked complex (Figure 3A) highlighted that **TSA-5** fit into a smaller cavity (Figure 2C) with an estimated surface area of 113.47 Å^2^, surrounded by the residues His125, Tyr127, Arg198, Ala200, His203, Arg311, Arg312, Ser313, Trp465, Ile467, and Gln469. Positively charged side chains of the residues Arg311 and Arg312 formed polar interactions with the electronegative F-atom and π–cation interactions with the aromatic ring of **TSA-5**. Although ^310^RRR^312^ was positioned very close to **TSA-5** in the docked structure, the side chain of Arg311 was most adjacent to forming a π–cation interaction with the aromatic ring of **TSA-5**. The unique presence of the F-atom supports the notion of significant polar interactions that leverage a stronger binding preference for **TSA-5** over candidate **TSA-4**. Additionally, it was observed from the docked complex that **TSA-5** makes three H-bonds with the protein **MbtB_Cy** (Figure 3B). The side-chain -OH of Ser313 forms a H-bond with the N-atom of **TSA-5**. The -OH group of **TSA-5** forms two H-bonds with the backbone atoms of Gly470 in the docked structure. The carbonyl oxygen plays the role of an acceptor, whereas the amide group of Gly470 shares its -H with the oxygen atom of **TSA-5**. **MbtB_Cy** residue Arg493 also forms favorable π–cation interactions with **TSA-5**’s aromatic ring. The residue Val473 forms a stable hydrophobic interaction with **TSA-5** and thus contributes to the formation of a stable binding of **TSA-5** at the active site pocket of **MbtB_Cy**. From these findings, we can hypothesize that the spatial geometry of the binding pocket is enough to accommodate structurally compact chemotypes but nonflexible enough to adjust the ‘Long-Linear-Tail’ of **TSA-1**, **2**, and **3**, which might plausibly be the reason for their inability to bind to the target enzyme. 

**Figure 4:**
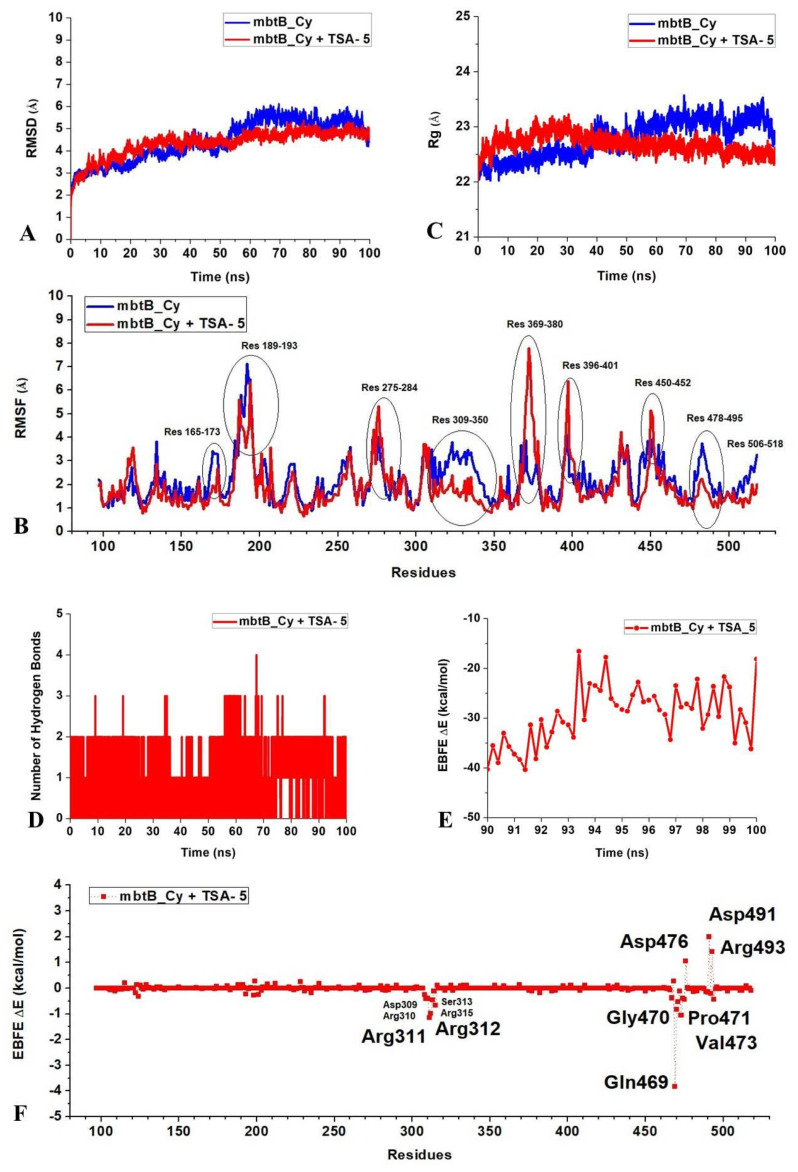
MD simulations indicated the formation of a stable complex between **TSA-5** and **MbtB_Cy**. (**A**) RMSD analyses showed that the backbone atoms of **MbtB_Cy** were stable while free and bound to **TSA-5**. (**B**) R_g_ analyses revealed that after binding with **TSA-5**, **MbtB_Cy** assumed a slightly more closed structure. (**C**) Higher RMSF values indicated that residues 369–380 were more mobile, whereas residues 309–350 and 478–495 were more confined after binding to **TSA-5**. (**D**) The number of hydrogen bonds formed between **TSA-5** and **MbtB_Cy** indicated the formation of a stable complex. (**E**) The estimated binding free energy (EBFE ΔE) obtained from the MMPBSA analyses indicated the formation of a stable **MbtB_Cy**-**TSA-5** complex. (**F**) Residue decomposition of binding free energy (EBFE ΔE) indicated that the residues Arg311, Arg312, Gln469, Gly470, Pro471, and Val473 contributed significantly towards the formation of a stable complex between **MbtB_Cy** and **TSA-5**.

To check the stability of the docked complex between **MbtB_Cy** and **TSA-5**, we performed 100 ns molecular dynamics simulations of **MbtB_Cy** and **MbtB_Cy** docked with **TSA-5** using Gromacs software 2019.4 [21]. The root mean square deviation (RMSD) of the backbone atoms indicates that **MbtB_Cy** is stabilized while free and bound to **TSA-5** (Figure 4A). The backbone RMSD values also show that the backbone of **MbtB_Cy** is relatively more stable in the protein–ligand complex (average RMSD = 4.34 ± 0.60 Å) than in the apo-protein (average RMSD = 4.45 ± 0.92 Å). The radius of gyration (R_g_) is often calculated to measure the compactness of a protein. **MbtB_Cy-TSA-5** had a slightly more compact structure (average R_g_ = 22.69 ± 0.16 Å) than **MbtB_Cy** alone (average R_g_ = 22.79 ± 0.34 Å) (Figure 4B). Root mean square fluctuation (RMSF) values of a group of atoms derived from MD simulations are often used to exhibit their relative conformational flexibilities. The RMSF values obtained from the MD simulations indicate that the residues 275–284, 369–380, 396–401, and 450–452 of **MbtB_Cy** have higher mobilities in the ligand-bound state than in the apo-state (Figure 4C). However, in the **TSA-5**-bound state, the residues 165–173, 189–193, 309–350, 478–495, and 506–518 showed lowered RMSF values compared to the apo-protein, which suggests that the mobility of these residues is more constrained upon binding to **TSA-5**. The residues ^310^RRRS^313^ and ^469^QGP^471^ of **MbtB_Cy** were also shown to bind to **TSA-5,** and thus both docking and RMSF analyses showed an important role of these residues in **TSA-5**’s binding. The formation of H-bonds often contributes toward stable binding between a protein and a ligand. The number of intermolecular hydrogen bonds formed between **MbtB_Cy** and **TSA-5** was 0–2 (average ~1) during the simulation (Figure 4D), suggesting stable binding between these two molecules. Snapshots taken at various time points of the MD trajectory suggest that the residue Gln469 (apart from Ser313 and Gly470) of **MbtB_Cy** also contributes significantly towards binding by forming H-bonds with the N-atom of **TSA-5**. Furthermore, using the molecular mechanics Poisson Boltzmann surface area method, trajectories from the last 10 ns of the 100 ns simulations were used to calculate the estimated binding free energy (EBFE ΔE) with snapshots every 200 ps (Figure 4E) [22]. The total binding free energy (ΔE −29.30 ± 5.76 kcal/mol) suggested a stable complex between ligand **TSA-5** and protein of interest **MbtB_Cy**. 

In our MMPBSA analysis, the average values of van der Waals energy (E_vdW_) and electrostatic energy (E_elec_) were found to be −36.44 ± 2.25 kcal/mol and −22.14 ± 8.31 kcal/mol, respectively. This indicates that the electrostatic interactions, in addition to the H bonding, also strongly favor the binding of **TSA-5** to **MbtB_Cy**. The nonpolar solvation energy (ΔG_nonpolar_) was also negative (−4.10 ± 0.17 kcal/mol), which favors the formation of the **MbtB_Cy-TSA-5** complex. However, the average polar solvation energies (ΔG_polar_) worked against the formation of **MbtB_Cy-TSA-5** (33.38 ± 3.62 kcal/mol). Packing of the polar side chains in the buried hydrophobic pockets could be a reason for high ΔG_polar_ values. Residue-specific binding free energies were plotted to show the average contribution of **MbtB_Cy** residues toward binding to **TSA-5** (Figure 4F). This analysis indicated that the residues Asp476, Asp491, and Arg493 contribute to positive ΔG_binding_ whereas the residues Arg311, Arg312, Gln469, Gly470, Pro471, and Val473 of **MbtB_Cy** contribute strongly to negative ΔE_binding_. The burial of the charged side chains of Asp476, Asp491, and Arg493 in the hydrophobic environment also explains the high ΔG_polar_ values. Overall, analyses of the MD trajectories suggested a stable binding between **TSA-5** and **MbtB_Cy**.

### 2.4. Cytotoxicity Profile Evaluation

First, the cytotoxicity profile of the HIT candidate **TSA-5** was assessed on eukaryotic cell lines, such as murine macrophage RAW 264.7 cells and human monocytic THP-1 cells, using a previously published technique [11], in order to comprehend the therapeutic efficacy. The particular dose concentrations of **TSA-5** at which cell growth is 90% inhibited, and subsequently, the calculated selectivity index (SI), are provided in Table 2. The candidate, **TSA-5**, offered a suitable therapeutic window against Mtb (Table 2). 

### 2.5. **TSA-5** Affects the Intracellular Survival of Mycobacterium aurum

**Figure 5:**
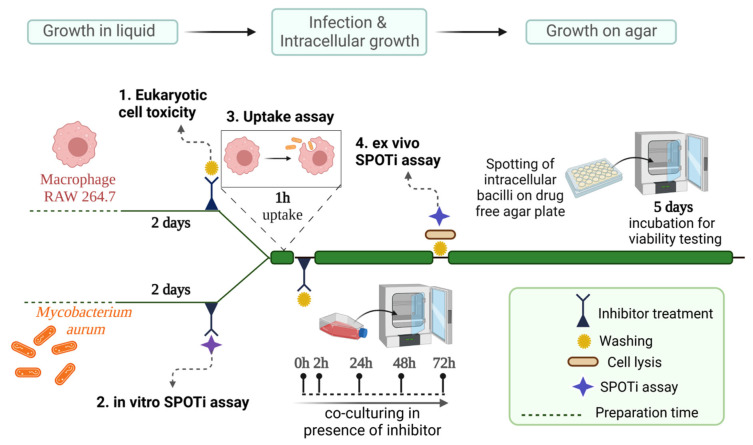
Schematic representation of *M. aurum*-infected macrophage surrogate model to investigate the antimycobacterial activity of compounds in vivo.

**Figure 6:**
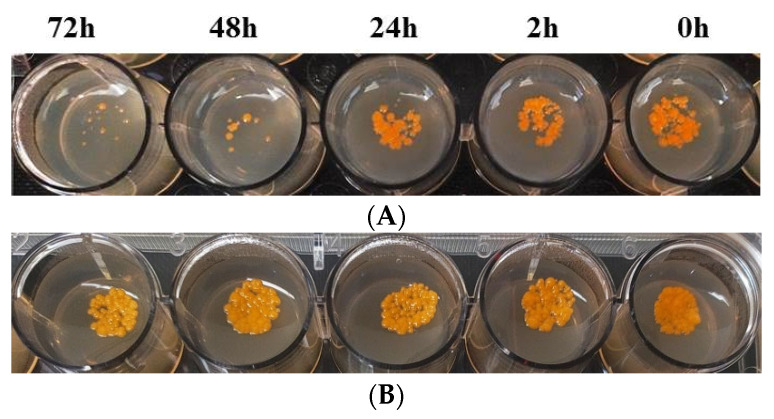
(**A**) TSA-5 kills more than 90% of intracellular surviving *M. aurum* in a macrophage RAW 264.7 infection model at 48 and 72 h using an 8 μg/mL dose concentration; in contrast, when there were no drug-treated wells (**B**), no intracellular *M. aurum* killing was observed.

In order to investigate the killing efficacy of the HIT candidate **TSA-5** on intracellularly surviving mycobacteria, a study was conducted employing an *M. aurum*-infected mouse macrophage model followed by a spot culture growth inhibition (SPOTi) assay [23,24]. This ex vivo infection paradigm replicates the micronutrient-poor host macrophage milieu where intracellularly surviving mycobacteria go through an early adaptation phase and strategically survive in the host immune system [25]. Using our previously published methodology [23], pre-established time points of 2 h, 12 h, 48 h, and 72 h were taken into consideration for the investigation (Figure 5) and we noted the reduction in *M. aurum*’s intracellular growth in the presence of the inhibitor **TSA-5** over the stipulated period. Experimental wells treated with no drugs were used as a negative control, showing no intracellular *M. aurum* killing. In this whole-cell phenotypic assay, **TSA-5** demonstrated more than 90% growth inhibition at the 48 h time point at the 8 μg/mL (MIC/4) dosage concentration, leading to a significant eight-fold increase in the antimicrobial selectivity index (Table 3 and Figure 6). Additionally, with hardly any variation from the 48 h killing pattern, a comparable pattern of intracellular growth suppression was also seen at the 72 h time point at the identical 8 μg/mL dosage concentration. According to the colony-forming unit (CFU), an 8 μg/mL dosage concentration of **TSA-5** administered over the course of 48 h is sufficient to kill more than 90% of intracellular mycobacteria compared to when there are no drug-treated wells (Figure 6). This significant finding indicates the mechanism of **TSA-5**’s enhanced intracellular killing compared to its in vitro antimycobacterial activity. 

### 2.6. Investigation of Efflux Pump Inhibition Profile of **TSA-5** against Mycobacterium smegmatis

To maintain the iron homeostasis in mycobacteria, extrusion of siderophores through MmpL4/MmpL5 is referred to as the critical mechanism [7]. Earlier studies reported that disruption of siderophore recycling leads to the accumulation of active siderophores inside the mycobacterial cells, resulting in self-poisoning via the Fenton reaction mechanism [7]. Based on this efflux-pump-mediated siderophore extrusion, we hypothesized that a pocket for siderophore binding should exist in the MmpL4/MmpL5 complex in mycobacteria. We hypothesized that this pocket should have a similar spatial geometry to that of the siderophore biosynthetic enzymes and intermediates, which accommodate the signature hydroxyphenyloxazoline moiety. As our compounds were designed based on the structure of hydroxyphenyloxazoline, we rationalized that the **TSA-5** might interfere with efflux pump MmpL4/MmpL5.

**Figure 7:**
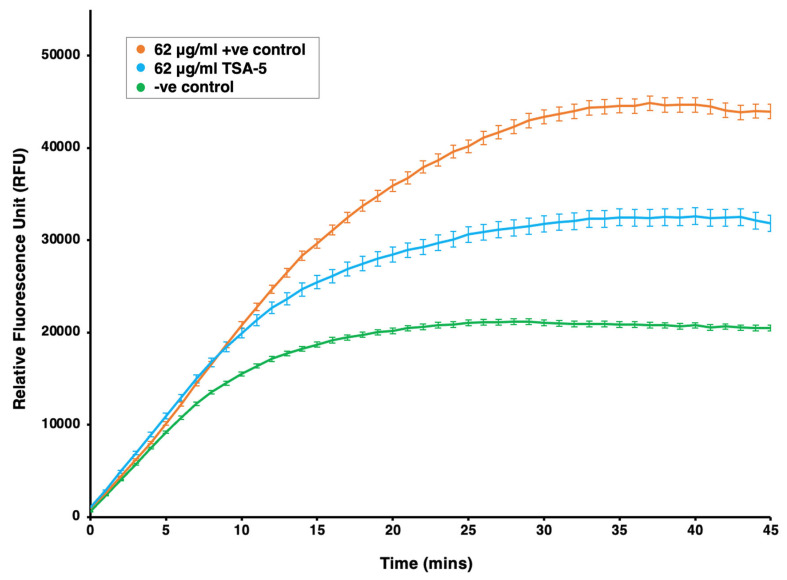
Accumulation of ethidium bromide (EtBr) in *M. smegmatis* cells over a 45 min period, in the presence of **TSA-5**, positive control (verapamil), and negative control (no inhibitor). Experiment was performed in biological triplicate (n = 3), and values represent the mean ± SD.

In this context, we employed an ethidium bromide (EtBr)-based fluorometric assay using *M. smegmatis* to assess the whole-cell efflux pump inhibitory activity [11]. Non-virulent mycobacterial strain, *M. smegmatis* mc2 155, is frequently employed as a surrogate model for the screening of recently identified efflux pump inhibitors in the early drug discovery stage [26]. The MIC_90_ of **TSA-5** against *M. smegmatis* in GAS and GAS-Fe medium were noted as 256 μg/mL and 256 μg/mL, respectively. A sub-MIC (MIC/4 dose concentration) of 62 μg/mL was used to investigate efflux pump inhibition of **TSA-5** against *M. smegmatis*. **TSA-5** demonstrated moderate efflux inhibition activity in *M. smegmatis* (Figure 7).

## 3. Materials and Methods

### 3.1. Materials and Instruments

Sigma-Aldrich (St. Louis, MI, USA) and Spectrochem (Mumbai, India) provided reagent-grade chemicals, which were used exactly as they were received. To check on the completion of the reaction, precoated Merck TLC plates that were readily available were utilized and UV light at 254 nm was used to check the results. All reactions were conducted under a nitrogen atmosphere. ^1^H NMR and ^13^C NMR spectra were captured on Bruker 400 and 100 MHz instruments, respectively. TMS was used as an internal standard. As a solvent, deuterated chloroform (CDCl_3_) was used. The unit of chemical shifts was δ ppm. Melting points (uncorrected) were measured in the capillary tubes of a hot-stage melting point apparatus that contained silicon oil. On a Bruker 100 MHz instrument, the spectra of DEPT-135 and DEPT-90 were recorded, and CDCl_3_ was employed as the solvent. On a Micromass Q-TOF Premier Tandem Mass Spectrometer, HRMS spectra were recorded. In this work, bacterial strains like *M. tuberculosis* H37Rv (ATCC 27294), *M. aurum* (ATCC 10437), *M. abscessus* (NCTC 13031), and *M. smegmatis* mc^2^ 155 (ATCC 700084) and eukaryotic cells like RAW 264.7 (ATCC-TIB71) and THP-1 (ATCC-TIB202) were used. Before performing the experiments, all the cultures underwent two passages, and they were all employed during the logarithmic growth phase. Minimal media such as glycerol–alanine salts (GAS) and ferric chloride (FeCl_3_)-supplemented glycerol–alanine salts (GAS-Fe) were used for MIC_90_ determination experiments. In the SPOTi agar plate method, agar was additionally supplemented with oleic acid–albumin–dextrose–catalase (OADC) enrichment (BD Difco^TM^), RPMI-1640 media, fetal bovine serum (FBS), and phosphate-buffered saline (PBS, Fisher Scientific, Hampton, NH, USA). Readings were taken on a BioTek Synergy 2 (Gen5) instrument. 

### 3.2. Chemical Synthesis

#### 3.2.1. Synthesis of Methyl (*E*)-3-Hydroxy-2-(((2-hydroxyphenyl)imino)methyl) Propanoate (**TSA-1**)

TSA-1 was synthesized via a simple one-pot reaction procedure. The condensation of salicylaldehyde (0.00163 mmol) with L-serine methyl ester hydrochloride (0.00196 mmol) in tetrahydrofuran medium resulted in the product methyl (*E*)-3-hydroxy-2-(((2-hydroxyphenyl)imino)methyl)propanoate. The reaction was catalyzed by magnesium sulphate (0.002 mmol) and triethylamine (0.00359 mmol) and an inert nitrogen environment was maintained throughout the reaction. 

#### 3.2.2. General Procedure for the Synthesis of Azomethines, **TSA-(2–5)**

Azomethines were synthesized via a simple one-pot reaction procedure. The condensation of *o*-hydroxy acetophenone (0.0014 mmol) with respective amines (pentan-1-amine for **TSA-2**, hexan-1-amine for **TSA-3**, phenylmethanamine for **TSA-4**, 4-fluorophenylmethanamine for **TSA-5** in 0.0028 mmol) in ethanol at room temperature, with stirring for 12 h in an inert nitrogen environment, resulted into azomethines. 

### 3.3. In Vitro Antimycobacterial Activity

The resazurin microtiter assay (REMA) was used to assess the MIC of TSA analogues on *M. tuberculosis*, *M. aurum*, *M. smegmatis*, and *M. abscessus* strains of pathogens, as reported earlier [11]. Briefly, REMA assays were conducted in a 96-well plate format (Corning Inc., Corning, NY, USA). Compounds were dissolved in DMSO (dimethyl sulfoxide) to a concentration of 10 mg/mL and further serially diluted in 2-fold dilutions. The compound concentration range of 256–0.5 µg/mL was used for the experiments in both GAS and GAS-Fe media. Compounds were tested in biological triplicate. RIF and INH were used as the positive controls and a 1–0.001 µg/mL concentration range was used in the experiments. To perform them, 10 mg/mL stock solution of compound or control was taken (10.24 μL) and added into the first well with 189.76 μL medium (total volume is 200 μL), followed by a 2-fold serial dilution, and next, we added 100 μL of bacteria of an early- to mid-log-growth mycobacterial strain with a 10^5^ colony forming unit (CFU)/mL culture concentration. Following 2-fold dilution of the drug concentration, the total volume was 200 μL in each well and the drug concentration was 256 µg/mL for the first well. Particularly, in assays for slow-growing *M. tuberculosis* and *M. aurum* strains, 200 μL of sterile deionized water was added to all outer-perimeter wells of plates to minimize the evaporation of medium from the test wells during the longer incubation period. The plates were then sealed with parafilm and incubated in static conditions at 37 °C for 8 days (*M. tuberculosis*), 72 h (*M. aurum* and *M. abscessus*), and 24 h (*M. smegmatis*). After the incubation period, 20 μL of resazurin solution (0.01% *w*/*v*) was added to the wells and the plates were again incubated at 37 °C for 24 h, 12 h, and 4 h for the *M. tuberculosis*, *M. aurum*, *M. abscessus*, and *M. smegmatis* strains, respectively. Cell viability was measured through the colorimetric change of resazurin (blue) to resorufin (pink) at an excitation of 530 nm and emission of 590 nm. TSI values were then calculated using the following formula:

$$\mathrm{TSI}\;(\mathrm{Target Specificity Index})=\frac{{MIC}_{90}\;in\;GAS-Fe\;medium}{{MIC}_{90}\;in\;GAS\;medium}$$

### 3.4. Cytotoxicity Activity

The cytotoxicity profile was assessed on confluent murine macrophage RAW 264.7 cells and human monocyte THP-1 cells using a resazurin assay in a 96-well plate format, according to a previously published protocol [11]. To do so, 2 μL of a 50 mg/mL stock solution of the compound was diluted in 200 μL of RPMI-1640 medium in the first row of a 96-well microplate, and 2-fold dilutions were performed along the rows, leaving the last row as a media control. **MTX** was used as a positive control and a 10–0.01 µg/mL concentration range was used in the experiments. In the next step, 100 μL culture solution containing 5 × 10^5^ cells/mL of RAW 264.7 and THP-1 cells in the logarithmic growth phase was added to each well of a 96-well plate, and then the plates were incubated for 48 h in a humidified CO_2_ (5%) incubator at 37 °C. After the incubation period, plates were washed twice with 1× PBS. Undifferentiated THP-1 cells are nonadherent, and so to avoid large cell loss between washes, the plates were centrifuged (1200 rpm, 5 min, using a Thermo Scientific 75003624 M-20 rotor, in a Heraeus Megafuge 16R centrifuge). Following that, 170 μL freshly prepared RPMI-1640 medium (supplemented with 10% FBS) was added to each well of the plates. Finally, freshly prepared 0.01% resazurin solution (30 μL) was then added to each well and the plates were further incubated under the above-mentioned conditions overnight (16 h). Experiments were performed in biological triplicate. At the end, after the overnight incubation, color change (blue to pink) was observed, followed by fluorescence intensity measured at λ_exc_ 560/λ_emi_ 590 nm. Next, the selectivity index (SI) was calculated.

$$\mathrm{SI}\;(\mathrm{Selectivity Index})=\frac{Cell\;Inhibition\;Concentration}{{MIC}_{90}\;in\;GAS\;medium}$$

### 3.5. Antimycobacterial Activity in M. aurum-Infected Murine Macrophage Model

The intracellular survival assay was performed using an *M. aurum*-infected RAW 264.7 macrophage model in a 24-well plate format according to our earlier published protocol [23]. In the assay, macrophage cells (5 × 10^5^ cells/mL) were infected with intracellularly surviving *M. aurum* to achieve a 10:1 multiplicity of infection (MOI) and then incubated in a 24-well plate for 1 h at 37 °C in a humidified incubator with 5% CO_2_. After the infection stage, the culture was washed three times with RPMI-1640 media, followed by incubation for 0–72 h (0 h, 2 h, 24 h, 48 h, and 72 h) with different sub-MIC concentrations of **TSA-5** in freshly prepared complete RPMI (RPMI-1640 medium containing 10% FBS). After the stipulated incubation period, cells were washed twice with RPMI-1640 media and lysed in 500 μL of sterile distilled water for 10 min, applying mechanical force using a sterile syringe plunger at room temperature (RT). In the next step, lysed cells were centrifuged at 16,000× *g* and RT for 10 min and then resuspended in 50 μL of sterile distilled water. Finally, 5 μL of the 50 μL cell suspension was spotted onto the wells of a 24-well plate containing 2 mL MB 7H10 agar, supplemented with 10% OADC. Plates were incubated at 37 °C for 5 days, and then plates were checked for the growth of bacterial colonies to assess the intracellular survival of *M. aurum*. The negative control comprised no drug-treated wells, showing no intracellular killing of *M. aurum* inside the model macrophages over the period of 72 h.

### 3.6. Whole-Cell Drug Efflux Pump Accumulation Assay against M. smegmatis

The assay was performed using early log-phase cells of *M. smegmatis,* and cells were grown in Middlebrook 7H9 broth (BD Difco^TM^) supplemented with 10% albumin–dextrose–catalase (ADC; Merck, Darmstadt, Germany) in a rotating incubator at 180 rpm and 37 °C [11]. Once the cultures reached OD_600_ ~1.0 (~10^8^ bacteria/mL), they were adjusted to OD_600_ 0.5 for the assay. All compounds were tested at a sub-MIC concentration (62 μg/mL) to ensure unaltered cell viability. Cells were collected through centrifugation and resuspended in an equivalent volume of 1× PBS and 0.05% Tween80 (Merck). The bacterial culture was aliquoted in PBS and 0.05% Tween80, assessed for the test compounds, and supplemented with 0.4% glucose as the source of energy for efflux pump activity. A stock of 50 μg/mL ethidium bromide (EtBr) was used as the substrate for efflux pumps; verapamil was used as the positive control. The assay was conducted in triplicate on a 96-well plate, with each assay conducted at the same time. The plate was read in a fluorimeter (Agilent BioTek Synergy™ 2 Multi-Mode Microplate Reader (Fisher Scientific UK Ltd., Loughborough, UK)) and programmed with the following parameters: excitation 540 nm, emission 620 nm, fluorescence gains 80, and the cycle of measurement every minute for a total period of 45 min at 37 °C.

### 3.7. Molecular Docking Studies

AutoDock 4.2 software [20] was used for the docking study. The Swiss-Model server (https://swissmodel.expasy.org/interactive accessed on 1 December 2023) was used to obtain the homology model of MbtB-Cy [17]. Nonpolar H-atoms were merged, and Gasteiger charges were assigned to the protein structure. The PRODRG webserver (http://davapc1.bioch.dundee.ac.uk/cgi-bin/prodrg accessed on 1 December 2023) was used to prepare the ligand structure. Blind docking was carried out covering the entire protein using a Lamarckian genetic algorithm (LGA) with 50 runs, 150 populations, 2,500,000 evaluations, and 27,000 generations.

### 3.8. Molecular Dynamics and MMPBSA Calculations

GROMACS 2019.4 software was used to carry out the molecular dynamics (MD) simulations using the OPLS-AA all-atom force field [27] and TIP4P water model. The box dimensions used to maintain the periodic boundary condition, and the total number of ions needed to mimic the physiological concentration of 0.16 M NaCl, were calculated using PACKMOL [28]. A minimum distance from any atom to the boundary of the box was 1 nm. Avogadro 1.2.0 software [29] was used to add hydrogens to the **TSA-5** structure. The LigParGen webserver [30] was used to obtain the charge parameters of **TSA-5**. 39 Na^+^, and 32 Cl^−^ ions were added to neutralize the system. Energy minimizations were performed using the steepest descent algorithm with a maximum force F_max_ less than 1000 kJ mol^−1^ nm^−1^. Two consecutive 100 ps simulations with NVT ensembles and NPT ensembles were carried out to equilibrate the system at 300 K and 1 bar pressure. MD simulations were run for 100 ns using a 2 fs time step and 1.2 nm long-range cut-off. PyMOL [31] was used to visualize the snapshots. Binding free energies of the protein–ligand system were calculated using the g_mmpbsa tool. The surface tension constant (γ) and the SASA constant for fitting were 0.0226778 kJ mol^−1^ Å^−2^ and 3.84928 kJ mol^−1^, respectively. Python scripts MmPbSaStat.py and MmPbSaDecomp.py [22] were used to calculate the binding energies and the residue-specific decomposition of the binding energies. Snapshots at an interval of 200 ps were collected from the last 10 ns of the MD simulation to calculate the MMPBSA binding free energies.

## 4. Conclusions

In this study, we investigated newly developed, rationally designed transition-state analogues (TSAs) with the distinctive *o*-hydroxyaromatic moiety of the mycobactin chemical framework, as new antitubercular agents. We explored the underlying fundamental chemical framework of azomethine, TSA analogues, and scaffold hopping enabled us to identify (*E*)-2-(1-((4-fluorobenzyl)imino)ethyl)phenol, **TSA-5**, as a new mycobactin biosynthesis inhibitor. From the findings, we deduced that HIT **TSA-5** has higher antitubercular activity in iron-deficient (GAS) than iron-enriched (GAS-Fe) medium, which suggests a target preference for mycobactin production suppression. Moreover, a spot culture growth inhibition (SPOTi) assay indicated the eradication of intracellularly surviving mycobacteria by **TSA-5**. An outline of **MbtB_Cy**’s active-site pocket was determined using homology structure prediction and Computed Atlas of Surface Topography of Protein investigations, which also revealed that **TSA-5** and **MbtB_Cy** had stable binding in molecular docking, MD analyses, and MMPBSA calculations. Intriguingly, the amino tail of the azomethine scaffold in **TSA-5** featured an electron-withdrawing F-atom, which helped to promote polar contacts with the residues Arg310, Arg311, and Arg312, and which facilitated the formation of a stable bonding highlighted in the in silico investigations. The results imply that the binding pocket present at the **MbtB_Cy** active site has a strong affinity for functional groups that pull electrons and significantly contribute to a stable polar interaction. Next, it would be exciting to explore the structural variety of the basic chemical building blocks using other electron-withdrawing substitutions such as Cl or Br for further analogues’ design and chemical synthesis. **TSA-5** extended a favorable spatial arrangement at the binding pocket of **MbtB_Cy** with many hydrogen bonds at the residues Ser313, Gln469, and Gly470, according to the MD analysis. **TSA-5** also demonstrated efflux inhibitory effects, allowing for future investigations of the substance’s potential adjunctive use in conjunction with other bactericidal agents though a detailed mechanism-of-action intervention. In sum, these research results should pave the way for expanding this chemical series and gaining further knowledge of the detailed molecular mechanism of the TSA chemical class of inhibitors.

## Figures and Tables

**Table 1:** Antitubercular activity of **TSA-**(**1**–**5**) analogues against *M. tuberculosis* H37Rv, *M. aurum*, and *M. smegmatis* strains of in GAS and GAS-Fe medium, and TSI determination.

Comp.	MIC_90_ μg/mL								
	*M. tuberculosis* (Mtb)			*M. aurum* (Ma)			*M. smegmatis* (Msmeg)		
	GAS	GAS-Fe	TSI	GAS	GAS-Fe	TSI	GAS	GAS-Fe	TSI
**TSA-1**	256	256	1	256	>256	>1	>256	>256	1
**TSA-2**	>256	256	nd	256	256	1	>256	>256	1
**TSA-3**	256	>256	>1	256	256	1	>256	>256	1
**TSA-4**	256	>256	>1	128	256	2	>256	>256	1
**TSA-5**	32	>256	>8	64	256	4	256	>256	>1
RIF	0.03	0.007	0.2	0.12	0.06	0.5	8	8	1
INH	0.03	0.005	0.2	0.02	0.01	0.5	2	2	1

Conc. for TSA derivatives was in the range of 256–0.5 μg/mL and for RIF and INH, conc. range was 1–0.001 μg/mL, respectively; ‘>’, when MIC conc. was obtained beyond the maximum limit (256 μg/mL) of test concentration; none of the analogues showed any growth inhibition against *M. abscessus* at the highest test concentration, and hence, they are not included in this table; nd, value not determined.

**Table 2:** Cytotoxicity profile evaluation and selectivity index (SI) determination of **TSA-5**.

Comp.	Cell-Line	GIC_90_ (μg/mL)	Mtb		Ma		Msmeg	
			MIC_90_	SI	MIC_90_	SI	MIC_90_	SI
**TSA-5**	RAW 264.7	256	32	8	64	4	256	1
	THP-1	256	32	8	64	4	256	1
**MTX**	RAW 264.7	2.50	nd	nd	nd	nd	nd	nd
	THP-1	0.31						

Growth inhibition assays were performed by using the resazurin microtiter assay (REMA) method. (Mtb = *Mycobacterium tuberculosis*; Ma = *Mycobacterium aurum*; Msmeg = *Mycobacterium smegmatis*); nd = not determined; **MTX** = methotrexate, positive control.

**Table 3:** Determination of intracellular minimum inhibitory concentration of **TSA-5** against intracellularly surviving *M. aurum* inside RAW 264.7 cells.

Comp.	Intracellular MIC_90_ (μg/mL) against *M. aurum*	GIC_90_ (μg/mL) RAW 264.7	Intracellular SI	Extracellular SI
**TSA-5**	8	512	64	8

Bacterial growth inhibition assays were performed by using the SPOTi agar plate method.

## Data Availability

Datas are contained within the article.

## Supplementary Materials

The following supporting information can be downloaded at: https://www.mdpi.com/article/10.3390/molecules28248115/s1, Supplementary Figure S1: 1H-NMR, 13C-NMR, HRMS spectra **TSA (1–5)** Depts 135 and Depts 90 spectra of **TSA-1**; Supplementary Figure S2: Ramachandran Plot of **MbtB-Cy**; Supplementary Figure S3: Accumulation of ethidium bromide (EtBr) in *M. abscessus* in the presence of **TSA-5**.
