# Supplementary material for: Rationally Designed Novel Phenyloxazoline Synthase Inhibitors: Chemical Synthesis and Biological Evaluation to Accelerate the Discovery of New Antimycobacterial Antibiotics
Source: Molecules. 2023 Dec 15;28(24):8115. doi: 10.3390/molecules28248115 (PMC10745776; doi:10.3390/molecules28248115)
Supplement: Supplementary file 1 [file molecules-28-08115-s001.zip › molecules-2717824-supplementary.pdf]

Supporting Information

Rationally Designed Novel Phenyloxazoline Synthase Inhibitors: Chemical Synthesis and Biological Evaluation to Accelerate the Discovery of New Antimycobacterial Antibiotics

Mousumi Shyam[1,2], Gourab Bhattacharje[3], Chris Daniel[2], Amrendra Kumar[4], Pragya Yadav[4], Piyali Mukherjee[5], Samsher Singh[5,†], Amit Kumar Das[3], Narender Tadigoppula[4], Amit Singh[5], Venkatesan Jayaprakash[1]* and Sanjib Bhakta[2]*

[1]Department of Pharmaceutical Sciences & Technology, Birla Institute of Technology, Mesra, Ranchi 835215 (Jharkhand), India

[2]Mycobacteria Research Laboratory, School of Natural Sciences, Institute of Structural and Molecular Biology, Birkbeck, University of London, Malet Street, London WC1E 7HX, UK

[3]Department of Biotechnology, Indian Institute of Technology Kharagpur, Kharagpur 721302, India

[4]Division of Medicinal & Process Chemistry, CSIR-Central Drug Research Institute, Sector 10 Janakipuram Extension, Sitapur Road, Lucknow 226031, India

[5]Department of Microbiology and Cell Biology and Centre for Infectious Disease Research, Indian Institute of Science, CV Raman Avenue, Bangalore 560012, India

[#]Current address; Nanyang Technological University, Lee Kong Chian School of Medicine, Singapore 308232

*Correspondence; venkatessanj@bitmesra.ac.in; Tel: +91-9470137264; s.bhakta@bbk.ac.uk /sanjib.bhakta@ucl.ac.uk; Tel: +44-20 3926 3505

Table of Contents

Title	Page Number
^{1}H-NMR, ^{13}C-NMR, HRMS spectra TSA (1-5)<br>Depts 135 and Depts 90 spectra of TSA-1	2
Ramachandran Plot of MbtB-Cy	11
Accumulation of ethidium bromide (EtBr) in *M. abscessus* in the presence of **TSA-5**	12

Supplementary Figure S1. ^{1}H-NMR, ^{13}C-NMR, HRMS, Depts 135 and Depts 90 spectra of TSA (1-5):

All the newly synthesized compounds were characterized by ^{1}H-NMR, ^{13}C-NMR, and HRMS spectroscopic analysis. Compound TSA-1 is additionally characterized by Depts 90 and Depts 135 analysis.

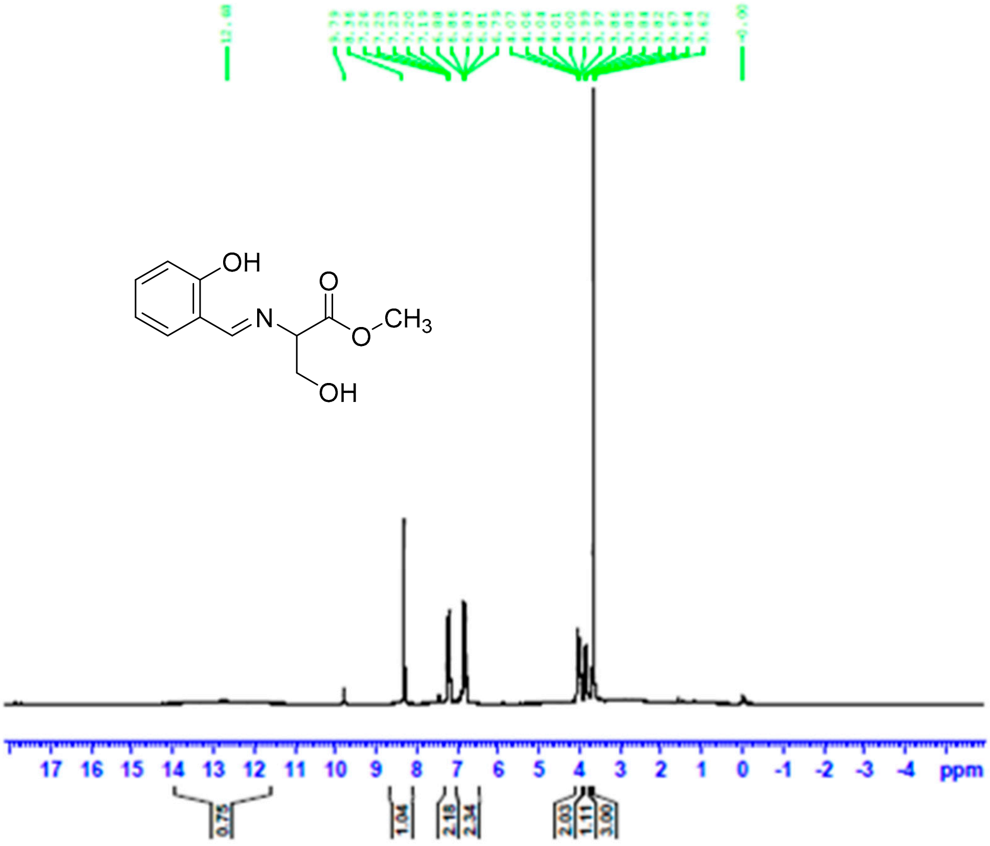

400 MHz ^{1}H-NMR spectrum of compound TSA-1 in $CDCl_3$

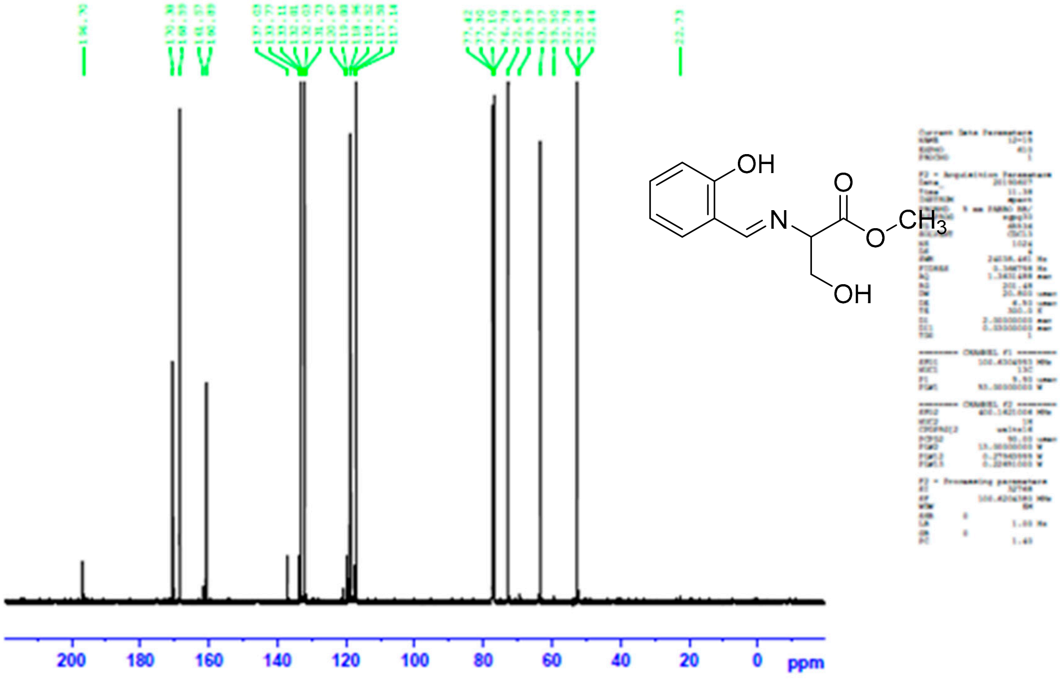

100 MHz ^{13}C-NMR spectrum of compound TSA-1 in $CDCl_3$

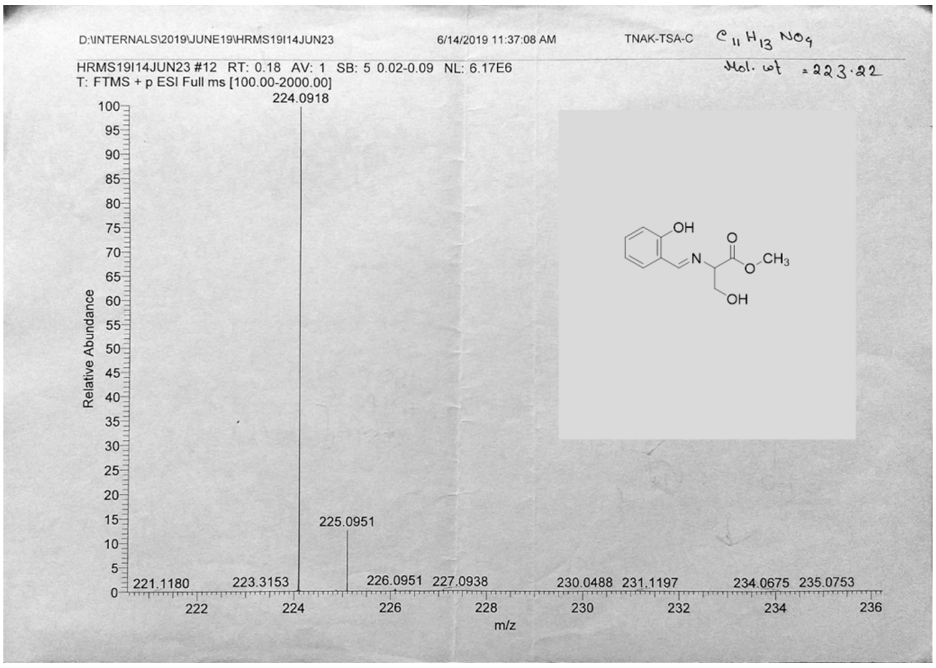

HRMS spectrum of compound TSA-1

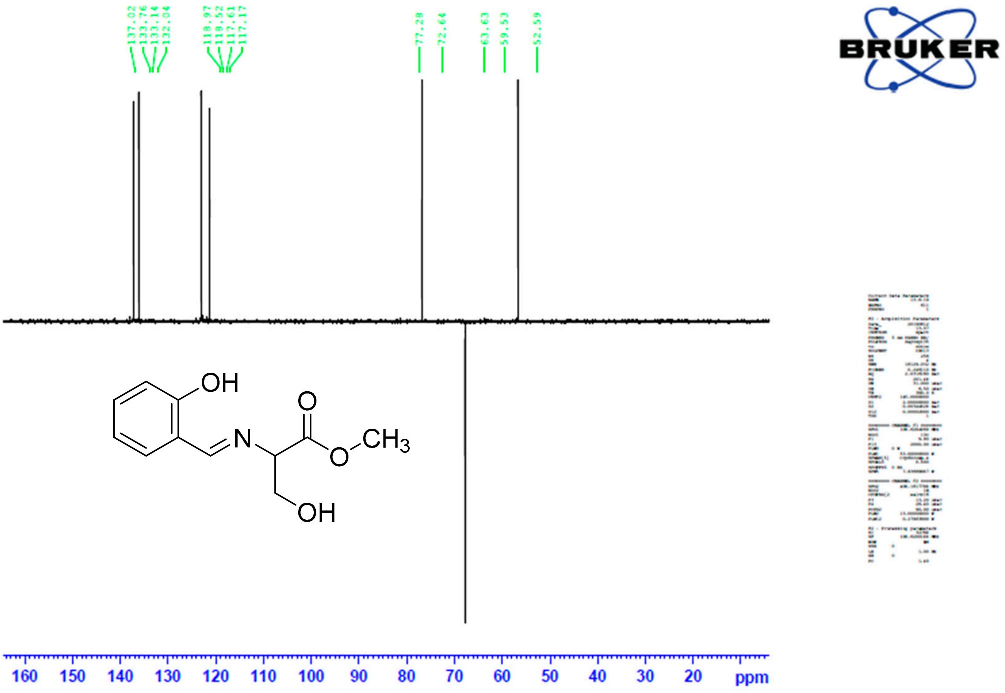

Depts-135 spectrum of TSA-1

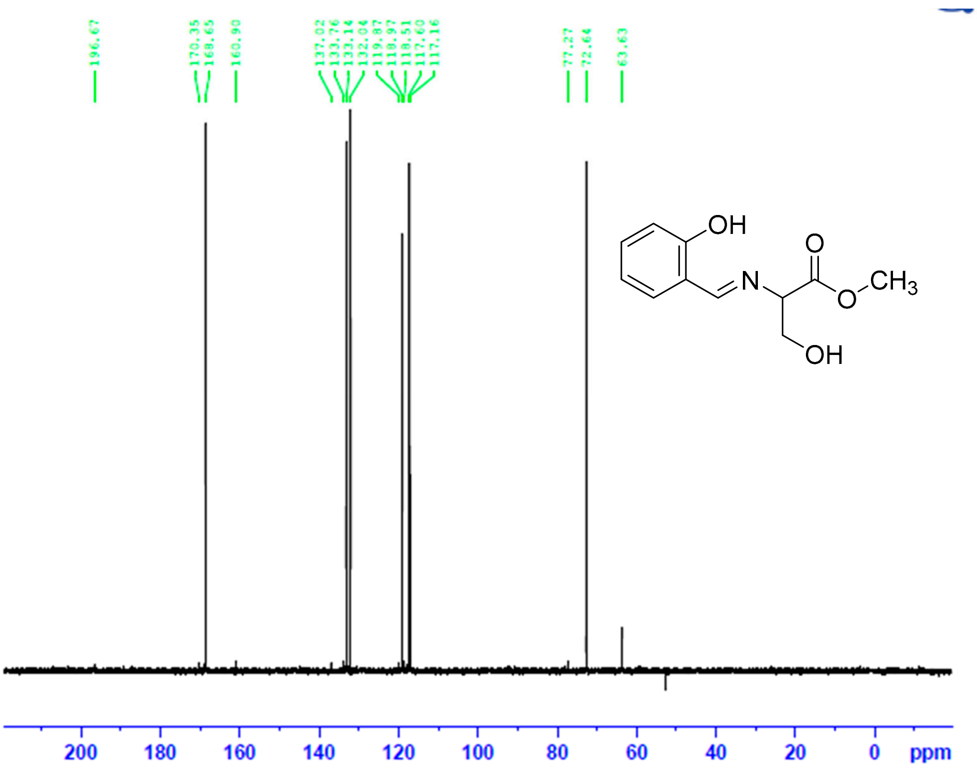

Depts-90 spectrum of TSA-1

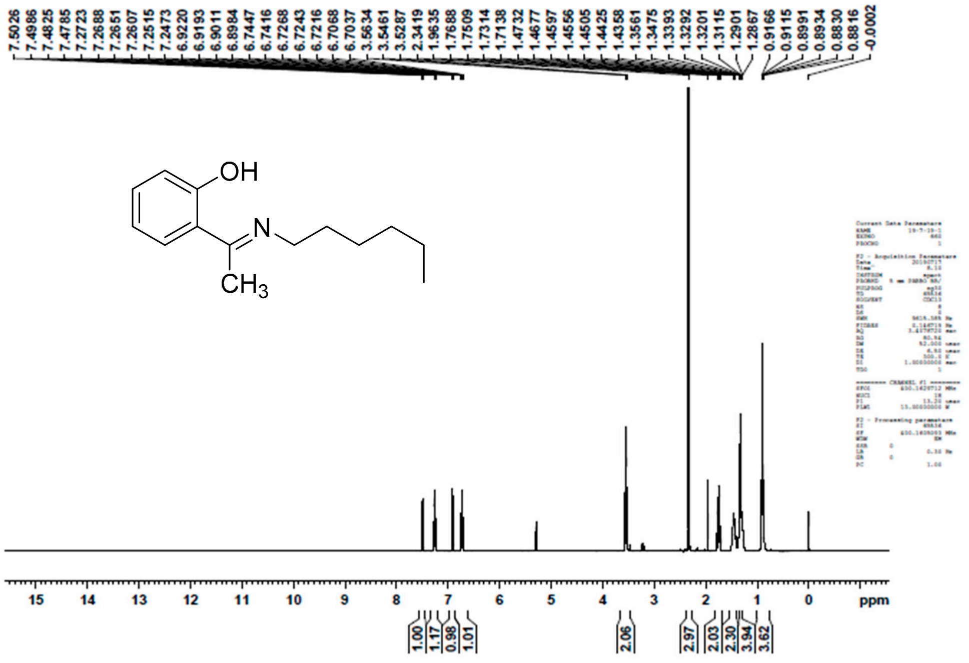

400 MHz ^{1}H-NMR spectrum of compound TSA-2 in $CDCl_3$

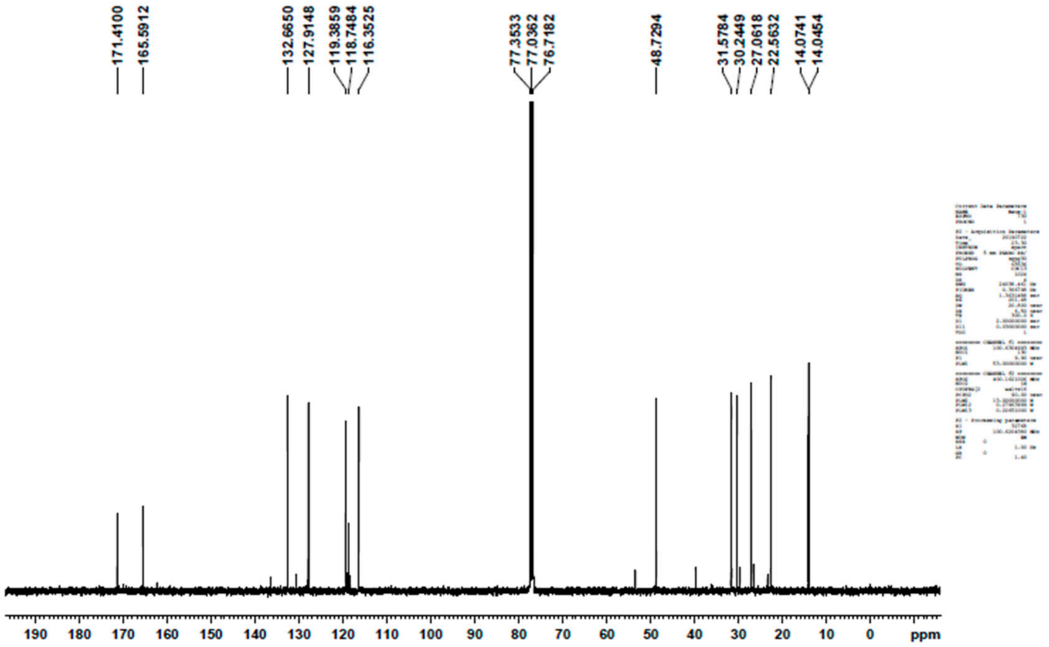

100 MHz ^{13}C-NMR spectrum of compound TSA-2 in $CDCl_3$

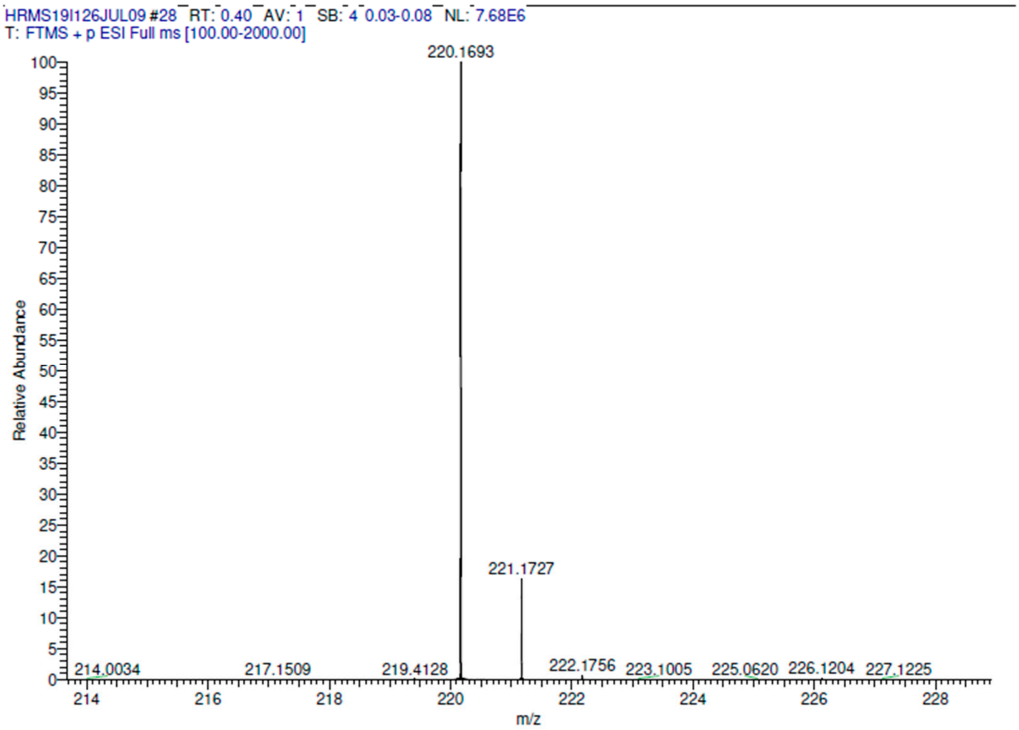

HRMS spectrum of compound TSA-2

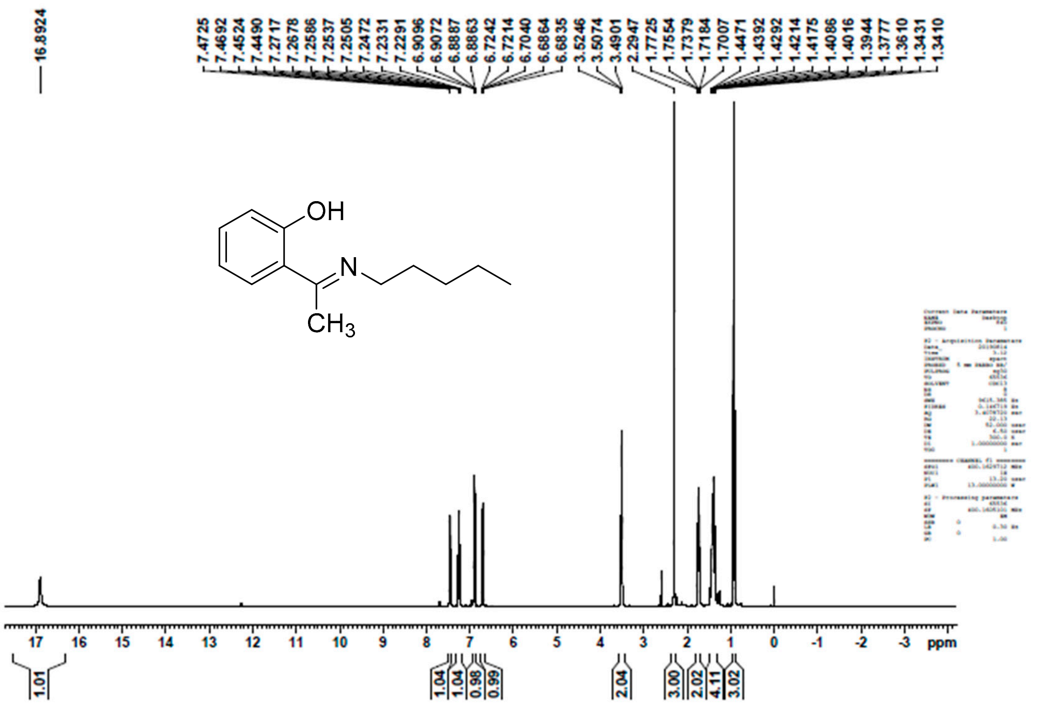

400 MHz ^{1}H-NMR spectrum of compound TSA-3 in $CDCl_3$

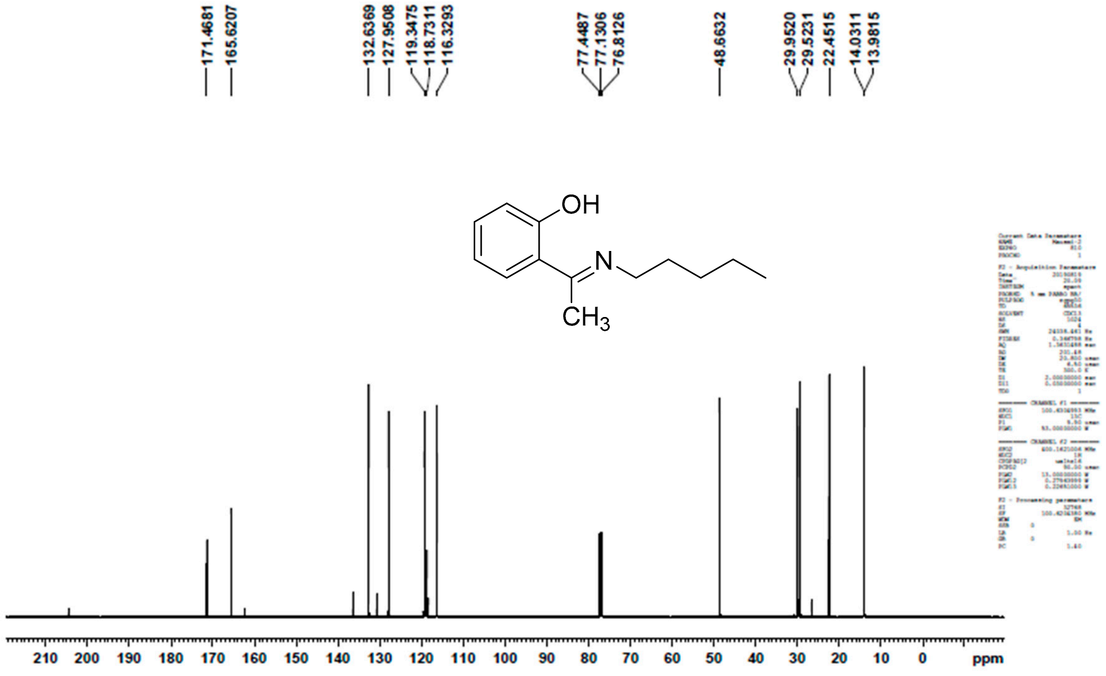

100 MHz ^{13}C-NMR spectrum of compound TSA-3 in $CDCl_3$

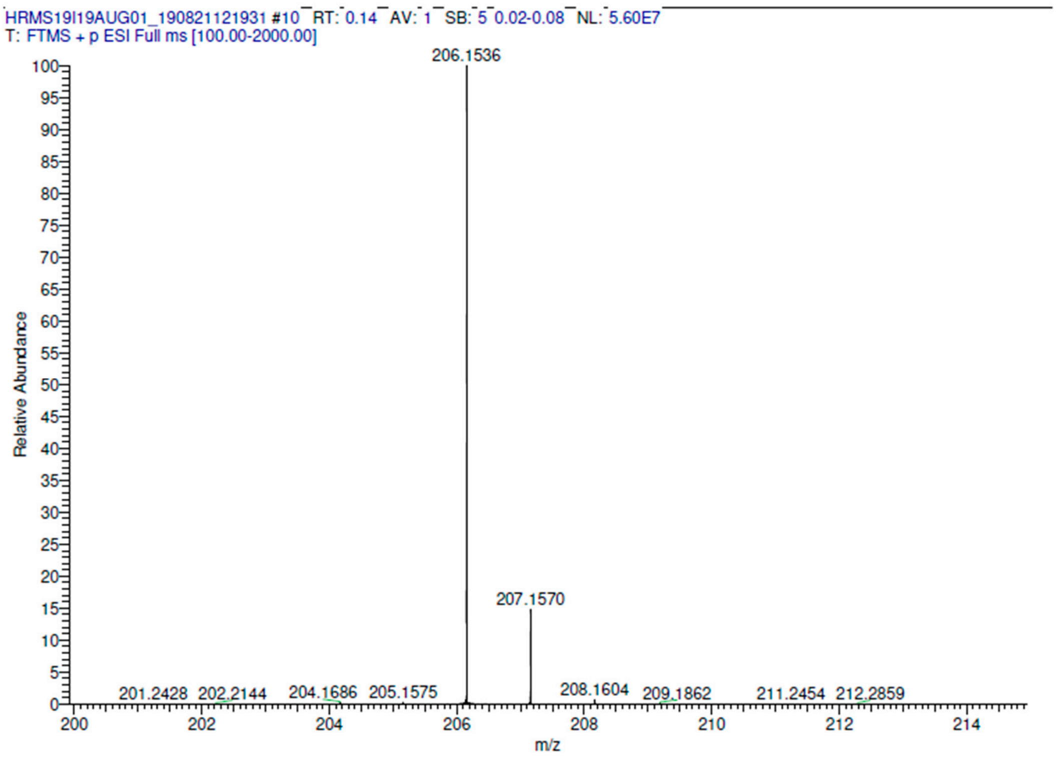

HRMS spectrum of compound TSA-3

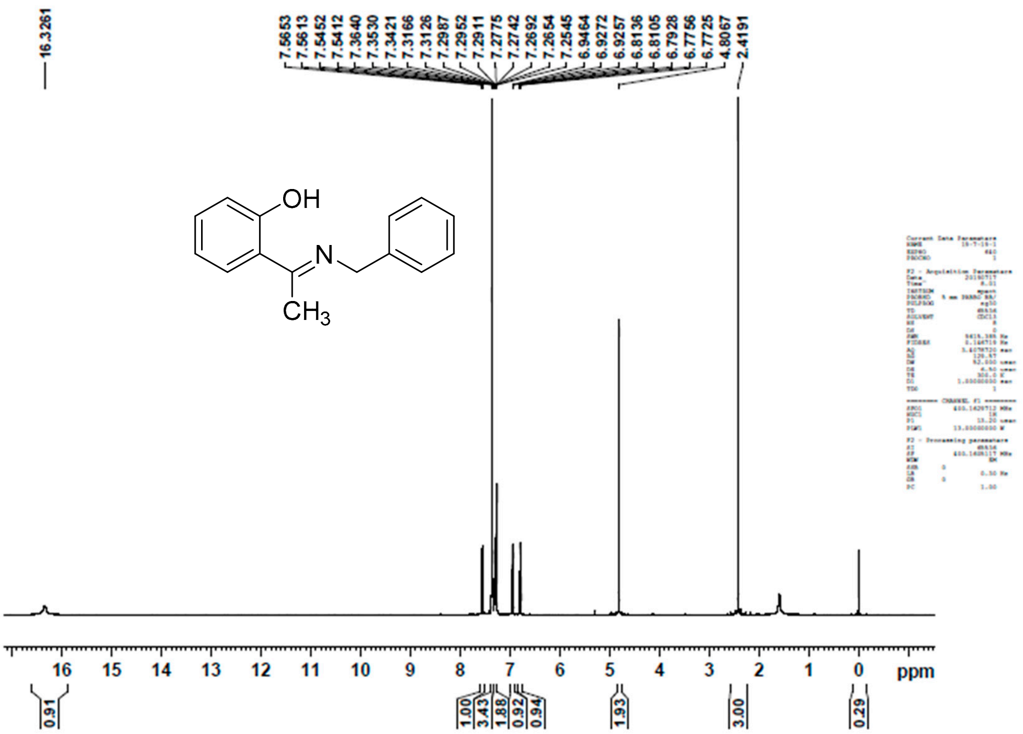

400 MHz ^{1}H-NMR spectrum of compound TSA-4 in $CDCl_3$

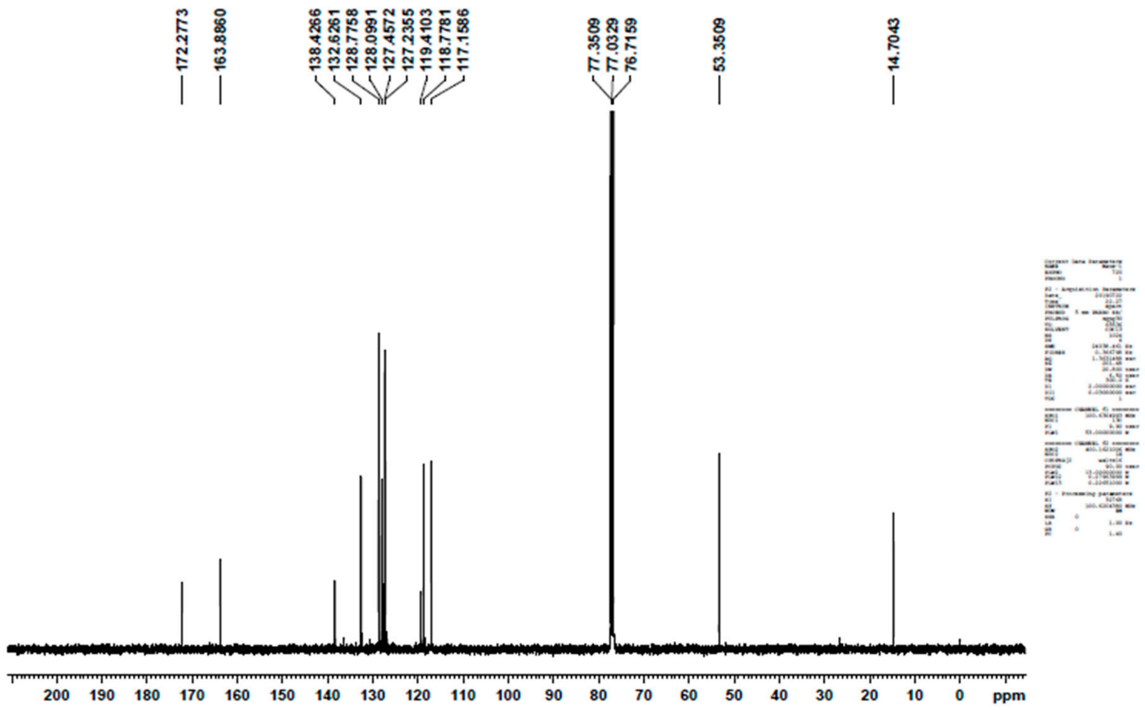

100 MHz ^{13}C-NMR spectrum of compound TSA-4 in $CDCl_3$

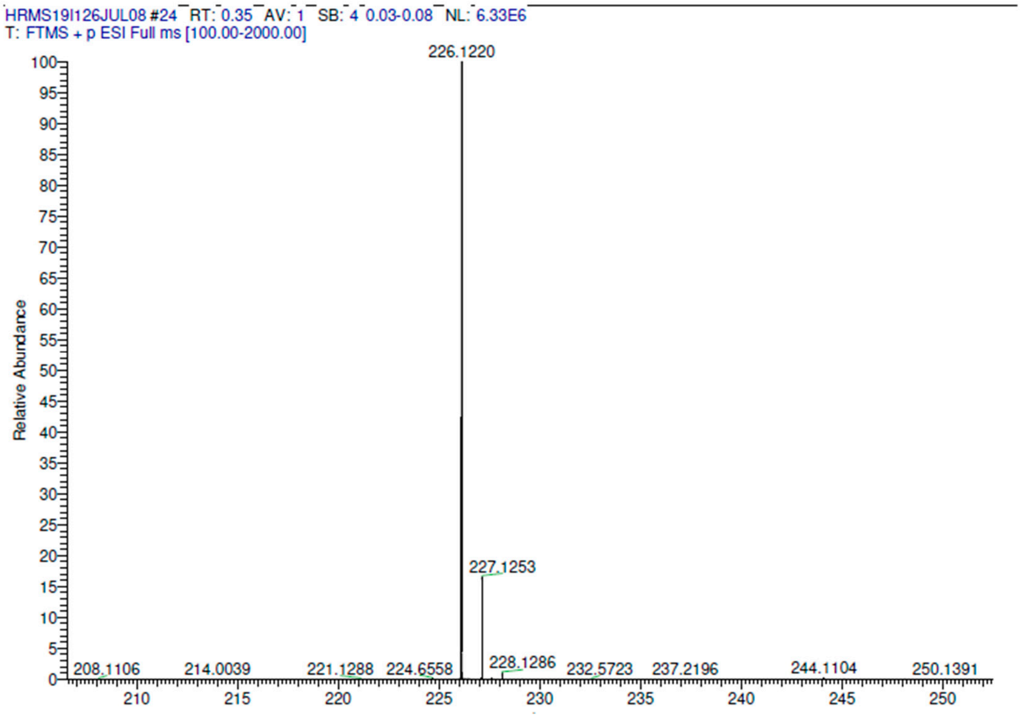

HRMS spectrum of compound TSA-4

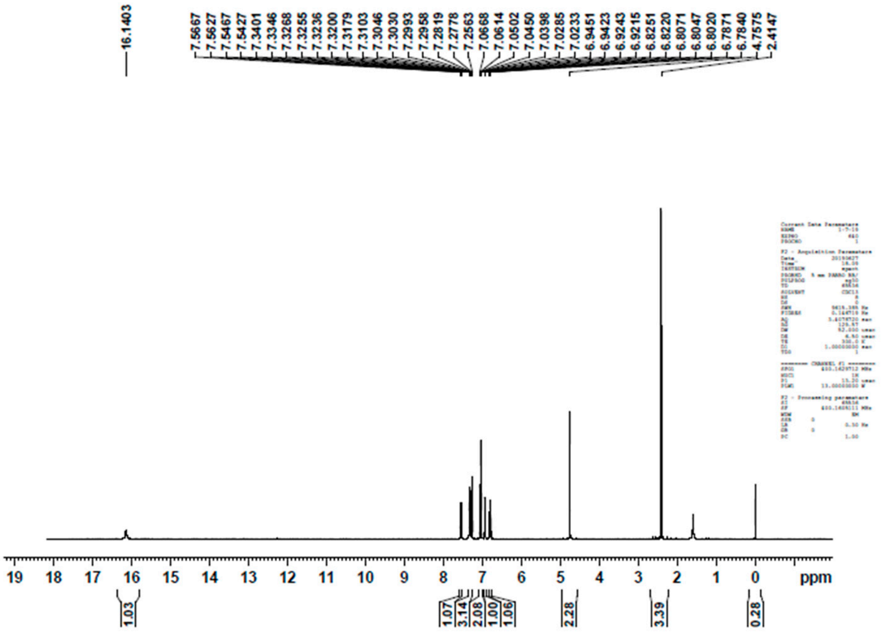

400 MHz ^{1}H-NMR spectrum of compound TSA-5 in $CDCl_3$

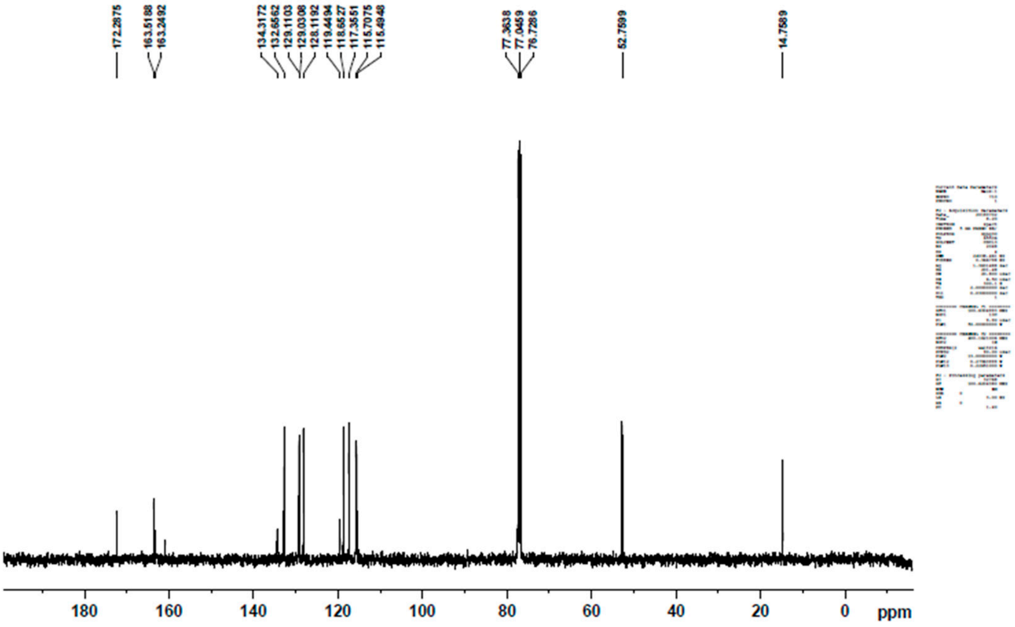

100 MHz ^{13}C-NMR spectrum of compound TSA-5 in $CDCl_3$

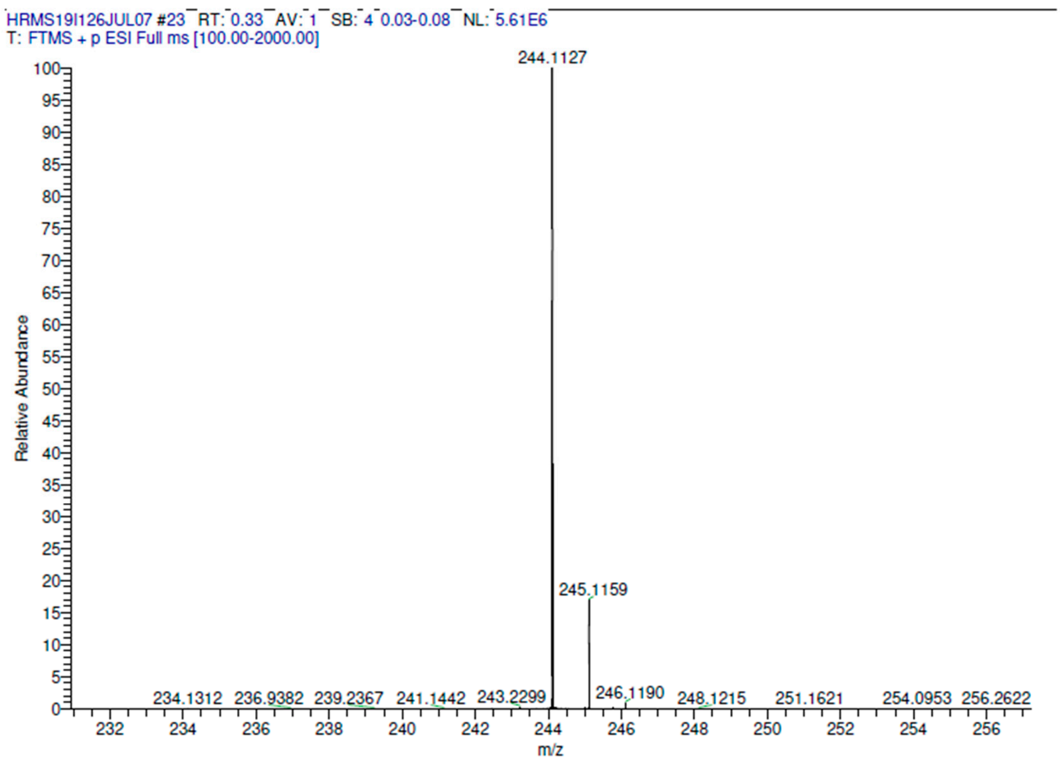

HRMS spectrum of compound TSA-5

1. **Supplementary Figure S1:** Ramachandran plot of the protein of interest, MbtB_Cy

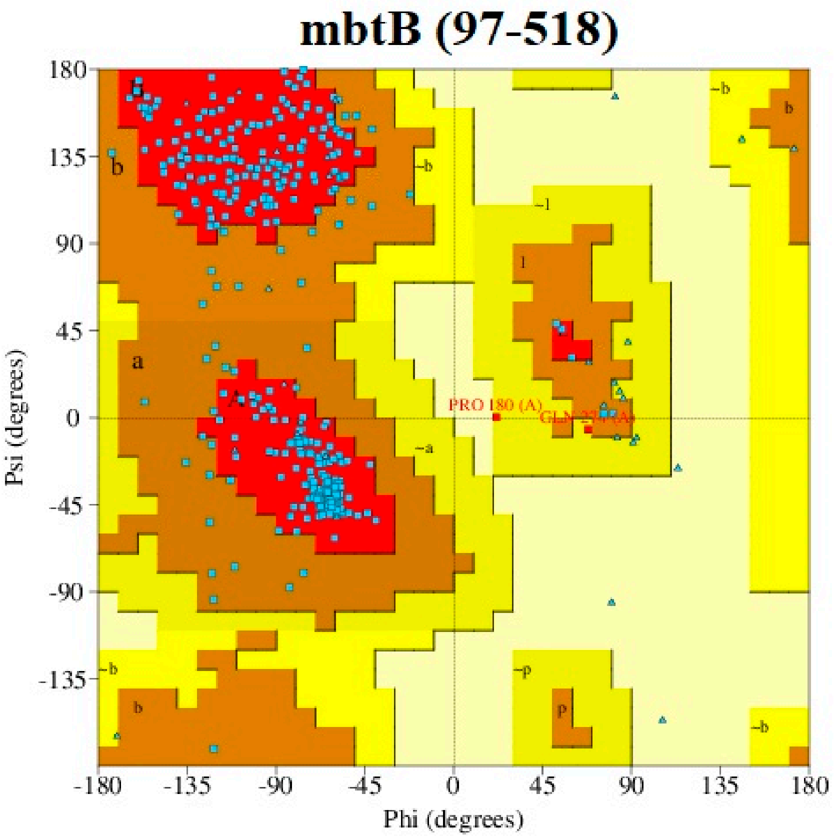

Supplementary Figure S2: Efflux-pump inhibition of TSA-5 against *M. abscessus*

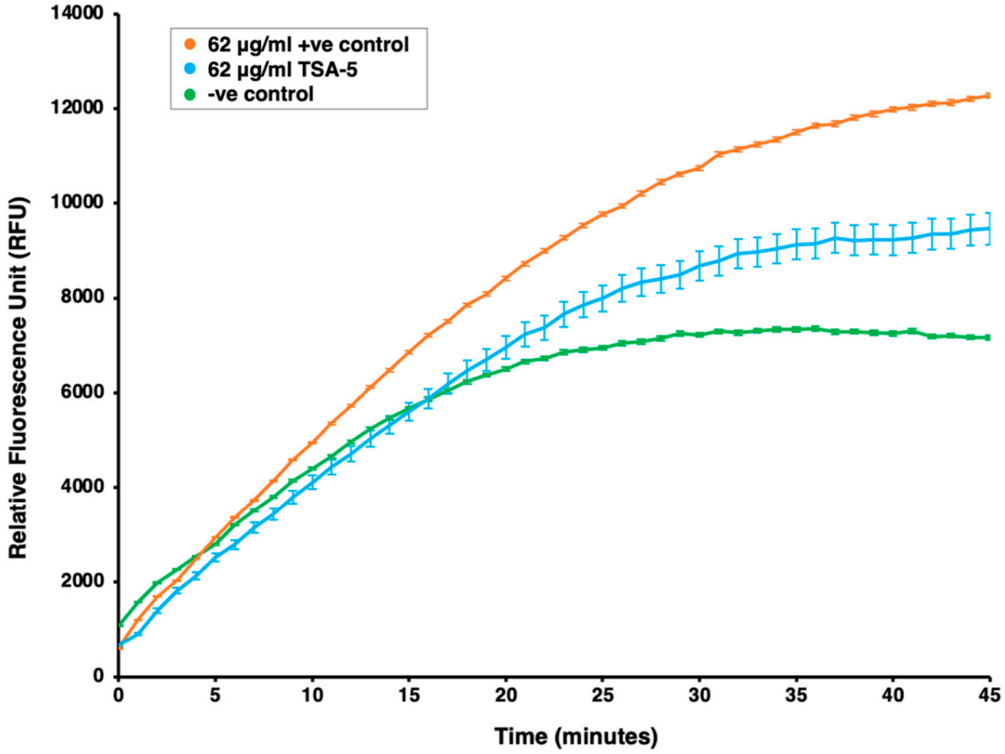

Supplementary Figure S3. Accumulation of ethidium bromide (EtBr) in *M. abscessus* cells over a 45-minute time frame, in the presence of **TSA-5**, positive control (verapamil), and negative control (no inhibitor). Experiment was performed in three biological replicates (n=3), values represent the mean ± SD.
